# The N6‐methyladenosine modification enhances ferroptosis resistance through inhibiting *SLC7A11* mRNA deadenylation in hepatoblastoma

**DOI:** 10.1002/ctm2.778

**Published:** 2022-05-06

**Authors:** Li Liu, Jiangtu He, Guifeng Sun, Nan Huang, Zhixuan Bian, Chang Xu, Yue Zhang, Zhongqi Cui, Wenqiang Xu, Fenyong Sun, Chengle Zhuang, Qiuhong Man, Song Gu

**Affiliations:** ^1^ Department of Clinical Laboratory Shanghai Fourth People's Hospital School of Medicine Tongji University Shanghai China; ^2^ Department of Clinical Laboratory Shanghai Tenth People's Hospital School of Medicine Tongji University Shanghai China; ^3^ Department of Laboratory Medicine Shanghai Children's Medical Center School of Medicine Shanghai Jiao Tong University Shanghai China; ^4^ Department of Central Laboratory Shanghai Tenth People's Hospital School of Medicine Tongji University Shanghai China; ^5^ Colorectal Cancer Center Shanghai Tenth People's Hospital School of Medicine Tongji University Shanghai China; ^6^ Department of Gastrointestinal Surgery Shanghai Tenth People's Hospital School of Medicine Tongji University Shanghai China; ^7^ Department of Surgery Shanghai Children's Medical Center School of medicine Shanghai Jiaotong University Shanghai China

**Keywords:** ferroptosis, hepatoblastoma, IGF2BP1, m6A methylation, resistance, SLC7A11

## Abstract

**Background:**

Solute carrier family 7 member 11 (SLC7A11) is overexpressed in multiple human tumours and functions as a transporter importing cystine for glutathione biosynthesis. It promotes tumour development in part by suppressing ferroptosis, a newly identified form of cell death that plays a pivotal role in the suppression of tumorigenesis. However, the role and underlying mechanisms of SLC7A11‐mediated ferroptosis in hepatoblastoma (HB) remain largely unknown.

**Methods:**

Reverse transcription quantitative real‐time PCR (RT‐qPCR) and western blotting were used to measure SLC7A11 levels. Cell proliferation, colony formation, lipid reactive oxygen species (ROS), MDA concentration, 4‐HNE, GSH/GSSG ratio and cell death assays as well as subcutaneous xenograft experiments were used to elucidate the effects of SLC7A11 in HB cell proliferation and ferroptosis. Furthermore, MeRIP‐qPCR, dual luciferase reporter, RNA pulldown, RNA immunoprecipitation (RIP) and RACE‐PAT assays were performed to elucidate the underlying mechanism through which SLC7A11 was regulated by the m6A modification in HB.

**Results:**

SLC7A11 expression was highly upregulated in HB. SLC7A11 upregulation promoted HB cell proliferation in vitro and in vivo, inhibiting HB cell ferroptosis. Mechanistically, SLC7A11 mRNA exhibited abnormal METTL3‐mediated m6A modification, which enhanced its stability and expression. IGF2 mRNA‐binding protein 1 (IGF2BP1) was identified as the m6A reader of SLC7A11, enhancing *SLC7A11* mRNA stability and expression by inhibiting *SLC7A11* mRNA deadenylation in an m6A‐dependent manner. Moreover, IGF2BP1 was found to block BTG2/CCR4‐NOT complex recruitment via competitively binding to PABPC1, thereby suppressing *SLC7A11* mRNA deadenylation.

**Conclusions:**

Our findings demonstrated that the METTL3‐mediated *SLC7A11* m6A modification enhances HB ferroptosis resistance. The METTL3/IGF2BP1/m6A modification promotes *SLC7A11* mRNA stability and upregulates its expression by inhibiting the deadenylation process. Our study highlights a critical role of the m6A modification in SLC7A11‐mediated ferroptosis, providing a potential strategy for HB therapy through blockade of the m6A‐SLC7A11 axis.

## Graphical abstract

1. Upregulation of SLC7A11 promotes hepatoblastoma tumorigenesis by enhancing ferroptosis resistance.
2. METTL3/IGF2BP1/m6A modification enhances the SLC7A11 mRNA stability and expression via inhibiting the deadenylation in an m6A‐dependent manner.
3. The competitive binding of IGF2BP1 blocks PABPC1 from recruiting the BTG2/CCR4‐NOT complex, thereby suppressing the deadenylation of SLC7A11 mRNA.

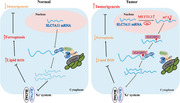

## 1 INTRODUCTION

Hepatoblastoma (HB) is a form of childhood liver cancer that is usually diagnosed in the first 3 years of life. It accounts for 28% of all liver tumours and two‐thirds of hepatic malignancies in the paediatric and adolescent population.^1^, ^2^ With recent advances in surgical resection and the combined application of chemotherapeutics, the 5‐year survival rate for HB patients has improved. However, the overall prognosis remains very poor for patients with unresectable and chemotherapy‐resistant tumours.^2^, ^3^ Therefore, exploring the underlying molecular mechanism of HB occurrence is of great significance for the development of new therapeutic strategies.

Ferroptosis is a newly identified iron‐dependent form of cell death, which is genetically, biochemically and morphologically distinct from necroptosis and apoptosis.^4^, ^5^ Ferroptosis is induced by the accumulation of lipid peroxidation products. Further, emerging evidence suggests that ferroptosis is a critical mechanism for tumour suppression.^6^, ^7^ Solute carrier family 7 member 11 (SLC7A11/xCT) is a catalytic subunit of system Xc^−^, which serves as a cystine/glutamate antiporter that transports extracellular cystine into cells. Cellular uptake of cystine leads to its rapid conversion to cysteine, which is a rate‐limiting precursor for glutathione (GSH) synthesis.^8^ Subsequently, glutathione peroxidase 4 uses this reduced GSH as a co‐factor to reduce lipid hydroperoxides to lipid alcohols, thereby protecting cells from lipid peroxidation‐induced ferroptosis.^9^ SLC7A11 is generally upregulated in tumour cells, especially those chemotherapy‐ and radiotherapy‐resistant tumour cells.^8^, ^10^ Suppressing the activity of SLC7A11 via genetic ablation or pharmacologic inhibition affects GSH synthesis, which leads to the accumulation of lipid peroxidation products, ultimately inducing ferroptosis in cancer cells.^5^, ^6^, ^7^, ^8^, ^11^ Thus, SLC7A11‐driven ferroptosis may represent a potential therapeutic target in cancer. However, the role and regulatory mechanisms of SLC7A11‐driven ferroptosis in HB remain unknown.

N6‐methyladenosine (m6A) is considered the most abundant mRNA modification in eukaryotic cells, occurring at the N6‐position of adenosine.^12^, ^13^ As one of the reversible mRNA modifications, m6A is regulated by the m6A ‘writer’ complex, m6A ‘erasers’ and m6A ‘readers’. The m6A ‘writer’ complex, acting as a methyltransferase, is composed of catalytic subunit methyltransferase‐like 3 (METTL3) and other subunits, including RBM15, Wilms tumour 1‐associated protein and METTL14 as well as KIAA1429, and so forth. M6A ‘erasers’, including alkB homologue 5 as well as fat mass and obesity‐associated protein (FTO), strip the m6A modification off‐target mRNAs. M6A ‘readers’, which bind and recognize the modification, determine the destiny of target mRNAs and include IGF2 mRNA‐binding proteins (IGF2BPs) as well as YT521‐B homology (YTH) domain family proteins.^12^, ^13^ The m6A modification regulates almost every aspect of RNA metabolism, including translation, export, decay, splicing, stabilization, as well as microRNA processing. Accumulating evidence shows that the m6A modification participates in carcinogenesis. For instance, it can regulate cell metastasis, proliferation, stem cell differentiation and homeostasis in cancer, representing a promising biomarker for cancer detection.^12^, ^13^, ^14^

In our previous study, we found that the m6A modification and METTL3 expression were significantly upregulated in HB. METTL3 promoted the proliferation of HB cells and was negatively correlated with the survival of HB patients, playing a role as an oncogene.^15^ In the current study, we demonstrate that *SLC7A11* mRNA is subjected to abnormal METTL3‐mediated m6A modification, which enhances its stability and expression, thereby promoting ferroptosis resistance in HB. Mechanistically, the m6A modification inhibits *SLC7A11* mRNA deadenylation through binding to the ‘reader’ protein IGF2BP1. Deadenylation is a rate‐limiting step of mRNA decay and is mainly catalyzed by the CCR4‐NOT complex in vertebrates.^16^, ^17^ It has been reported that the CCR4‐NOT complex can be recruited to the mRNA poly(A) tail by poly(A)‐binding protein 1 (PABPC1) to deadenylate mRNAs, or is directly recruited by YTHDF2 to destabilize m6A‐modified mRNAs.^18^, ^19^ Herein, we demonstrated that IGF2BP1 competitively binds to PABPC1 to block the recruitment of the BTG2/CCR4‐NOT complex, thereby suppressing the *SLC7A11* mRNA deadenylation.

## 2 MATERIALS AND METHODS

### 2.1 Human tissue samples and cell culture

HB and matched normal liver tissues were obtained from 35 patients who underwent surgical resection at the Shanghai Children's Medical Center. The tissues were quick‐frozen using liquid nitrogen upon resection and then stored at −80°C until RNA and protein extraction. The Medical Ethics Committee of Shanghai Children's Medical Center approved the research protocol and provided written informed consent.

Human HepG2, HuH6 and HEK293T cells were used in the current study. These three cell lines were obtained and cultured according to our previous work.^15^

### 2.2 M6A immunoprecipitation sequencing (MeRIP‐seq) and mRNA sequencing (mRNA‐seq)

MeRIP‐seq and mRNA sequencing (mRNA‐seq) were performed at Shanghai Cloud‐Seq Biotech Ltd. Co. (Shanghai, China) using an Illumina HiSeq 4000 sequencer (Illumina). The subsequent bioinformatics analyses were also carried out by the company.

The full MeRIP‐seq experimental procedure was described in our previous work.^15^ For mRNA‐seq, the detailed protocol is shown in Supporting Information S2.

### 2.3 In vitro assays and animal studies

The detailed protocols for cell transfection, lentivirus transduction, RNA isolation, RT‐qPCR, western blotting, Co‐IP, CCK8, colony formation, RNA pulldown, RIP, MeRIP‐qPCR, RACE‐PAT and dual‐luciferase reporter assays as well as animal experiments are shown in Supporting Information S2.

### 2.4 Measurements of MDA concentration and GSH/GSSG ratio

Lipid peroxidation assay kit (Abcam, #ab118970) was used to assess the malondialdehyde (MDA) concentration. GSH and GSSG assay kits (Solarbio, #BC1175 and #BC1180) were used to measure the GSH/GSSG ratio.

### 2.5 Measurements of lipid peroxidation and cell death

Lipid peroxidation and cell death were measured according to a published protocol.^20^ Briefly, treated cells were stained with BODIPY‐C11 (5 μM; Invitrogen) or propidium iodide (1:1000; Invitrogen) for 5 or 30 min at 37°C and were then analyzed via flow cytometry.

### 2.6 Detection of RNA stability

The stability of *SLC7A11* mRNA was assessed according to a published protocol.^21^ Briefly, HB cells with or without METTL3/IGF2BP1 knockdown were treated with actinomycin *D* with a final concentration of 5 μg/ml to terminate transcription and were then collected at 0, 30, 60 and 120 min, respectively. RNA was extracted from the cells and analyzed via RT‐qPCR.

### 2.7 Statistical analysis

All experiments were made in triplicate, and GraphPad Prism 7 software (GraphPad Software) was used to analyze the data. All data are expressed as the mean ± standard deviation (SD). The significance of differences between the two groups was determined using the Student's *t*‐test, and one‐way analysis of variance was used to compare multiple groups. Differences between groups were considered statistically significant when *p < .05, **/##*p* < .01, ***p < .001, or ****p < .0001.

## 3 RESULTS

### 3.1 SLC7A11 is upregulated in HB tissues

**FIGURE 1:**
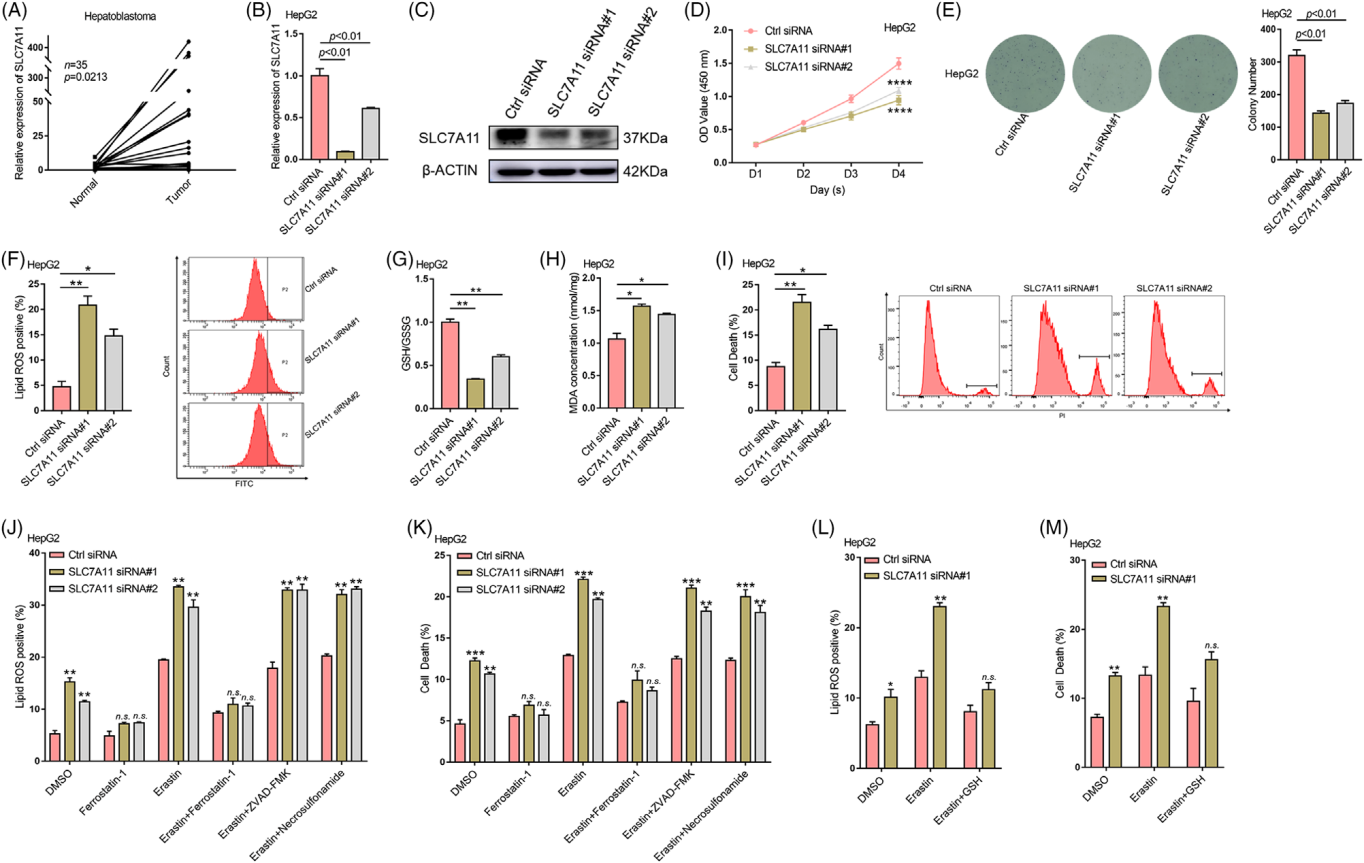
Solute carrier family 7 member 11 (SLC7A11) promotes proliferation and mediates ferroptosis in HepG2 cells. (A) Relative expression of SLC7A11 in human HB and matched normal tissues was determined via RT‐qPCR (*n* = 35). HepG2 cells were transfected with siRNAs targeting SLC7A11, and silencing efficiency was verified via RT‐qPCR (B) and western blotting (C). HepG2 cells transfected with *SLC7A11* siRNA#1 or *SLC7A11* siRNA#2 were subjected to CCK8 assays (D) and colony formation assays (E) to evaluate the role of SLC7A11 in HB cell viability and proliferation. (F) HepG2 cells were treated with *SLC7A11* siRNA#1 or *SLC7A11* siRNA#2 for 48 h, and lipid ROS levels were then measured via BODIPY C11 staining coupled with flow cytometry. The relative GSH/GSSG ratio (G) and malondialdehyde (MDA) concentration (H) in HepG2 cells, which were transfected with *SLC7A11* siRNA#1 or *SLC7A11* siRNA#2 for 48 h, were detected using GSH, GSSG, and lipid peroxidation assay kits, respectively. (I) HepG2 cells were treated with *SLC7A11* siRNA#1 or *SLC7A11* siRNA#2 for 60 h, and cell death was then determined via propidium iodide (PI) staining coupled with flow cytometry. HepG2 cells transfected with *SLC7A11* siRNA#1 or *SLC7A11* siRNA#2 were treated with DMSO, ferrostatin‐1, erastin, erastin + ferrostatin‐1, erastin + ZVAD‐FMK, or erastin + necrosulfonamide, respectively, and lipid ROS levels (J) as well as cell death (K) were then determined via flow cytometry. HepG2 cells transfected with *SLC7A11* siRNA#1 or *SLC7A11* siRNA#2 were treated with DMSO, erastin, or erastin + GSH, respectively, and the levels of lipid ROS (L) as well as cell death (M) were then determined via flow cytometry. Erastin: 30 μM, Ferrostatin‐1: 1 μM, ZVAD‐FMK: 10 μM, Necrosulfonamide: 1 μM, GSH: 0.8 mM. Ctrl, control. All quantitative data are shown as the mean ± SD from three independent experiments. n.s., no significant difference, *p < .05, **p < .01, ***p < .001, ****p < .0001

As previously mentioned, ferroptosis is genetically, morphologically and biochemically distinct from other forms of cell death, and it plays an important role in cancer biology.^22^ However, related research in HB is still very limited. To this end, an mRNA‐seq analysis of five pairs of HB and normal tissues was performed. The abundance of various mRNAs was dramatically altered in tumour tissues compared to normal tissues (Figure S1A). Volcano and scatter plots displayed the variation in mRNA expression between these two groups (Figure S1B,C). In total, we identified 4308 mRNAs that were differentially expressed (fold change > 2.0, *p* < .05). Compared to normal tissues, 2868 mRNAs were upregulated, and 1440 mRNAs were downregulated in HB tissues. The Kyoto Encyclopedia of Genes and Genomes pathway analysis was performed to analyze these two groups of differentially expressed mRNAs. Figure S1D,E shows the top 10 enriched terms, respectively. Notably, SLC7A11, a key amino acid transporter in ferroptosis, was found to be significantly upregulated (Figure S1F). Subsequently, we verified the expression of SLC7A11 in HB and matched normal tissues using RT‐qPCR and western blotting. Its expression was markedly elevated in HB tissues (Figure 1A; Figure S1G). The significant upregulation of SLC7A11 expression in HB indicates that it might be involved in HB progression.

### 3.2 SLC7A11 mediates HB cell ferroptosis

To examine the biological role of SLC7A11 in HB, two small interfering RNAs (siRNAs) were used to silence SLC7A11 in HepG2 and HuH6 cells (Figure 1B,C, Figure S2A,B). CCK8 and colony formation assays (Figure 1D,E, Figure S2C,D) indicated that silencing SLC7A11 considerably inhibited cell proliferation in both cell lines. SLC7A11 acts as a component of the system Xc^−^ cystine/glutamate antiporter, is highly upregulated in human tumours, and can protect cells from lipid peroxidation‐induced ferroptosis.^5^, ^6^, ^7^ Thus, we assessed the levels of lipid ROS, the GSH/GSSG ratio, MDA concentration, 4‐HNE and cell death after silencing SLC7A11 in HepG2 and HuH6 cells. SLC7A11 deficiency significantly increased lipid ROS levels, the MDA concentration, 4‐HNE levels and cell death in HepG2 as well as HuH6 cells, while decreasing the GSH/GSSG ratio (Figure 1F–I; Figure S2E–I). Moreover, the ferroptosis inhibitor ferrostatin‐1 reversed the increase in lipid ROS levels and cell death induced via SLC7A11 knockdown (Figure 1J,K, Figure S2K,L). In addition, SLC7A11 deficiency enhanced the ferroptosis sensitivity induced by erastin, a ferroptosis inducer that inhibits the function of system Xc^−^ cystine/glutamate antiporter.^23^ This enhancement could be rescued by ferrostatin‐1 or GSH treatment but not by necroptosis inhibitor necrosulfonamide or apoptosis inhibitor ZVAD‐FMK (Figure 1J–M; Figure S2J–L). Our results indicated that SLC7A11 mediates HB cell ferroptosis. Next, we overexpressed SLC7A11 in HepG2 and HuH6 cells and subjected them to CCK8, colony formation and flow cytometry assays. The results demonstrated that SLC7A11 upregulation significantly enhanced the proliferation and ferroptosis resistance of HB cells (Figures S3). Taken together, SLC7A11 plays an oncogenic role in HB cells and enhances ferroptosis resistance in vitro.

### 3.3 SLC7A11 promotes HB tumour growth in vivo

To verify the role of SLC7A11 in HB tumorigenesis, nude mice (4‐week‐old male) were subcutaneously injected with sh‐SLC7A11 HuH6 cells or negative control (sh‐NC) HuH6 cells in order to establish a xenograft tumour model. RT‐qPCR analysis revealed a significant downregulation of SLC7A11 expression in sh‐SLC7A11 HuH6 cells compared to sh‐NC HuH6 cells (Figure S4A). The xenograft tumour model demonstrated that deletion of SLC7A11 dramatically suppressed tumour volume and weight (Figure S4B–D), suggesting that SLC7A11 could promote HB tumour growth in vivo. To examine the regulatory effect of SLC7A11 on HB ferroptosis sensitivity in vivo, we injected another 10 mice (five in each of the sh‐SLC7A11 and sh‐NC groups) intraperitoneally with imidazole ketone erastin (IKE), which is a metabolically stable, potent system Xc^−^ inhibitor and a ferroptosis inducer suitable for animal experiments.^28^ The results indicated that SLC7A11 knockdown largely enhanced IKE‐induced HB ferroptosis sensitivity (Figure S4G–I). Furthermore, the GSH/GSSG ratio and MDA concentration in xenograft tumours confirmed the inhibitory effect of SLC7A11 on HB ferroptosis (Figure S4E,F). Overall, these results supported the notion that SLC7A11 promotes HB tumorigenesis by enhancing ferroptosis resistance in vivo.

### 3.4 METTL3‐mediated m6A modification enhances the stability and expression of SLC7A*11* mRNA

**FIGURE 2:**
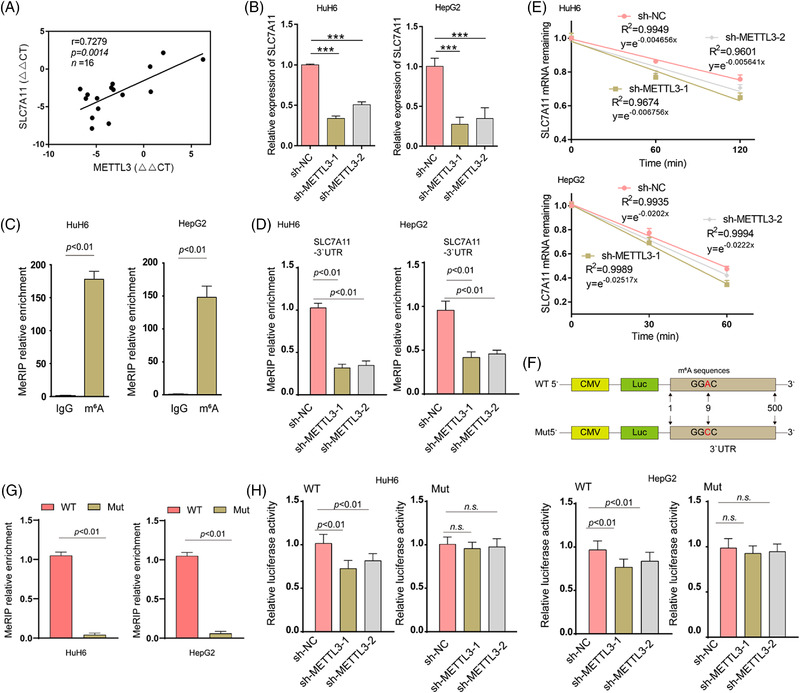
METTL3 mediates the m6A modification of *SLC7A11* mRNA, enhancing its stability and expression. (A) Pearson's correlation analysis indicated a positive correlation between METTL3 and SLC7A11 expression (*n* = 16) (*r*  =  0.7279, *p*  =  0.0014). The ΔΔCt values obtained from tumour‐normal tissue pairs (*n* = 16) were applied for Pearson's correlation analysis. (B) Relative expression of SLC7A11 in HuH6 and HepG2 cells upon METTL3 knockdown was determined via RT‐qPCR. (C–D) The relative m6A enrichment levels at the indicated site within the *SLC7A11* mRNA 3′UTR was verified via meRIP‐qPCR analysis in HuH6 and HepG2 cells with or without METTL3 knockdown. (E) The decay rate of *SLC7A11* mRNA in HuH6 and HepG2 cells with or without METTL3 depletion upon ActD (5 μg/ml) treatment was measured via RT‐qPCR at the indicated time points. (F) A schematic presentation of the pmir‐GlO luciferase reporters containing WT and Mut (GGAC to GGCC) *SLC7A11* mRNA 3′UTR. (G) MeRIP‐qPCR was used to verify m6A levels of WT and Mut pmir‐GlO plasmids. (H) Luciferase activities of the WT and Mut pmir‐GlO plasmids were measured in HuH6 and HepG2 cells with or without METTL3 knockdown. All quantitative data are presented as the mean ± SD from three independent experiments. WT, wide‐type; Mut, mutation; n.s., no significant difference; ***p < .001

Integration of the mRNA‐seq and meRIP‐seq data identified 70 upregulated genes with 75 high m6A methylation sites (these genes are listed in Table S3). Of note, SLC7A11 was one of the top overlapping genes identified in these two data sets. The m6A modification of *SLC7A11* mRNA was abnormally increased in HB tissues (Figure S5A–C). These results indicated that the upregulation of SLC7A11 might be modulated via m6A methylation. As shown in Figure 2A, there was a significant positive correlation between SLC7A11 and METTL3 expression levels in HB tissues. To further investigate the association between m6A methylation and SLC7A11 expression, we first determined the mRNA expression levels of SLC7A11 in METTL3 knockdown HepG2 and HuH6 cells. Knockdown of METTL3 significantly downregulated the expression of SLC7A11 (Figure 2B). Results from the MeRIP‐qPCR assay showed that *SLC7A11* mRNA could be enriched using an anti‐m6A antibody, and m6A modification near the putative m6A site was significantly reduced after METTL3 knockdown (Figure 2C,D). The regulation of mRNA stability is among the major functions of m6A methylation.^24^ Therefore, a pan transcription inhibitor, ActD, was used to treat HepG2 and HuH6 cells, in order to examine whether METTL3 regulates *SLC7A11* mRNA stability. We found that METTL3 depletion considerably reduced the half‐life of *SLC7A11* mRNA (Figure 2E). Finally, we examined whether METTL3 destabilizes *SLC7A11* mRNA via the putative m6A site. The WT *SLC7A11* 3′UTR and Mut *SLC7A11* 3′UTR (GGAC to GGCC) were cloned downstream of the Firefly luciferase encoding region in the pmir‐GLO vector (Figure 2F). MeRIP‐qPCR results showed that WT *SLC7A11* 3′UTR but not Mut *SLC7A11* 3′UTR was highly enriched (Figure 2G). Moreover, METTL3 knockdown reduced *SLC7A11* mRNA stability (Firefly luciferase activities represent mRNA stability) in HuH6 and HepG2 cells, while this reduction was not observed when the Mut reporter was introduced (Figure 2H). Taken together, our results indicated that the upregulation of SLC7A11 is mediated via the METTL3‐mediated m6A modification which enhances *SLC7A11* mRNA stability.

### 3.5 IGF2BP1 recognizes the m6A modification of *SLC7A11* mRNA, enhancing its stability and expression

**FIGURE 3:**
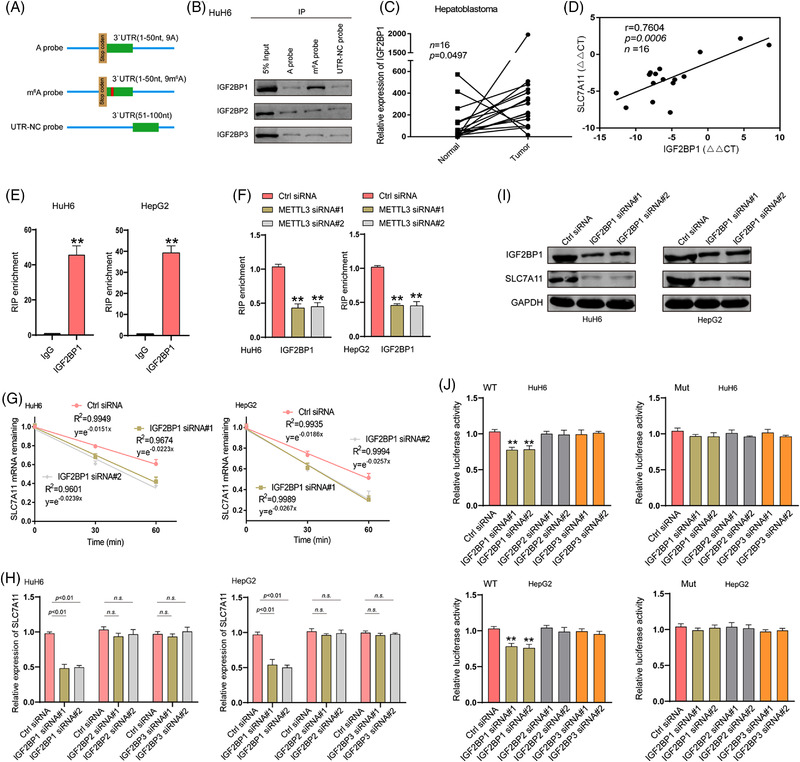
IGF2BP1 recognizes the m6A modification of *SLC7A11* mRNA and enhances its stability and expression. (A) A schematic presentation of the SLC7A11 probes with methylated or unmethylated adenosine for screening the m6A reader of SLC7A11. (B) RNA pulldown followed by western blotting for endogenous IGF2BP protein screening in HuH6 cell lysates incubated with synthetic SLC7A11 probes (A probe, m6A probe and UTR‐NC probe). (C) Relative expression of IGF2BP1 in human HB and matched normal tissues was determined via RT‐qPCR (*n* = 16). (D) The Pearson's correlation coefficient was used to evaluate the correlation between IGF2BP1 and SLC7A11 expression in HB tissues (*n*  =  16) (*r*  =  0.7604, *p*  <  .001). (E) RIP‐qPCR results show the association of *SLC7A11* 3′UTR with IGF2BP1 in HuH6 and HepG2 cells. (F) RIP‐qPCR was used to detect the binding of IGF2BP1 and *SLC7A11* 3′UTR in HuH6 and HepG2 cells upon METTL3 knockdown. (G) Reduced *SLC7A11* mRNA half‐life under IGF2BP1 silencing in HuH6 and HepG2 cells. Expression levels of *SLC7A11* mRNA and protein upon IGF2BP deletion were determined via RT‐qPCR (H) and western blotting (I). (J) Relative luciferase activities of WT and Mut pmir‐GlO reporters were measured in HuH6 and HepG2 cells with or without IGF2BP knockdown. IP, immunoprecipitation; Ctrl, control; WT, wide type; Mut, mutation. All quantitative data are presented as the means ± SD of three independent experiments. n.s., no significant difference; **p  <  .01

The fate of m6A‐modified mRNA is determined by m6A readers. To identify m6A readers acting on *SLC7A11* mRNA, an in vitro RNA pulldown assay was performed in HuH6 cells using synthesized partial *SLC7A11* 3′UTR probes. We found that IGF2BP1, but not IGF2BP2 and IGF2BP3, was pulled down by the *SLC7A11* 3′UTR probe with an m6A modification at the GGAC motif rather than by the probe without an m6A modification (Figure 3A,B). Subsequently, we determined IGF2BP1 expression in HB tissues and matched normal tissues via RT‐qPCR. Significantly elevated expression was observed in HB tissues, and SLC7A11 expression was positively correlated with IGF2BP1 expression (Figure 3C,D). These data indicated that IGF2BP1 might recognize and bind to the m6A modification of *SLC7A11* mRNA, thereby regulating its stability and expression. Next, RIP‐qPCR was performed to evaluate whether the m6A modification modulated the intracellular *SLC7A11* mRNA‐IGF2BP1 interaction. The results demonstrated that IGF2BP1 bound to the 3′UTR of *SLC7A11* mRNA in HepG2 and HuH6 cells. Based on METTL3 loss‐of‐function experiments in HuH6 and HepG2 cells, we found that the interaction of intracellular *SLC7A11* mRNA‐IGF2BP1 was determined by m6A levels (Figure 3E,F). Furthermore, RT‐qPCR and western blotting assays were performed to investigate whether IGF2BP1 could regulate *SLC7A11* mRNA stability and expression. IGF2BP1 downregulation decreased the stability and expression of SLC7A11 in HuH6 and HepG2 cells (Figure 3G–I). A dual‐luciferase assay further indicated that the stability of SLC7A11 was regulated by IGF2BP1 in an m6A‐dependent manner (Figure 3J). Notably, in addition to IGF2BP1, YTHDF2, YTHDF3 and YTHDC2 have also been reported to regulate the stability of m6A‐modifited mRNA.^24^ However, silencing either one of these did not reduce SLC7A11 expression in HepG2 and HuH6 cells. Silencing efficiency for IGF2BP2, IGF2BP3, YTHDF2, YTHDF3 or YTHDC2 was verified via RT‐qPCR (Figure S6A,B). These data suggested that IGF2BP1 preferentially binds to m6A‐methylated *SLC7A11* mRNA and stabilizes it in an m6A‐dependent manner.

### 3.6 IGF2BP1 inhibits *SLC7A11* mRNA deadenylation regulated by the CCR4‐NOT complex

**FIGURE 4:**
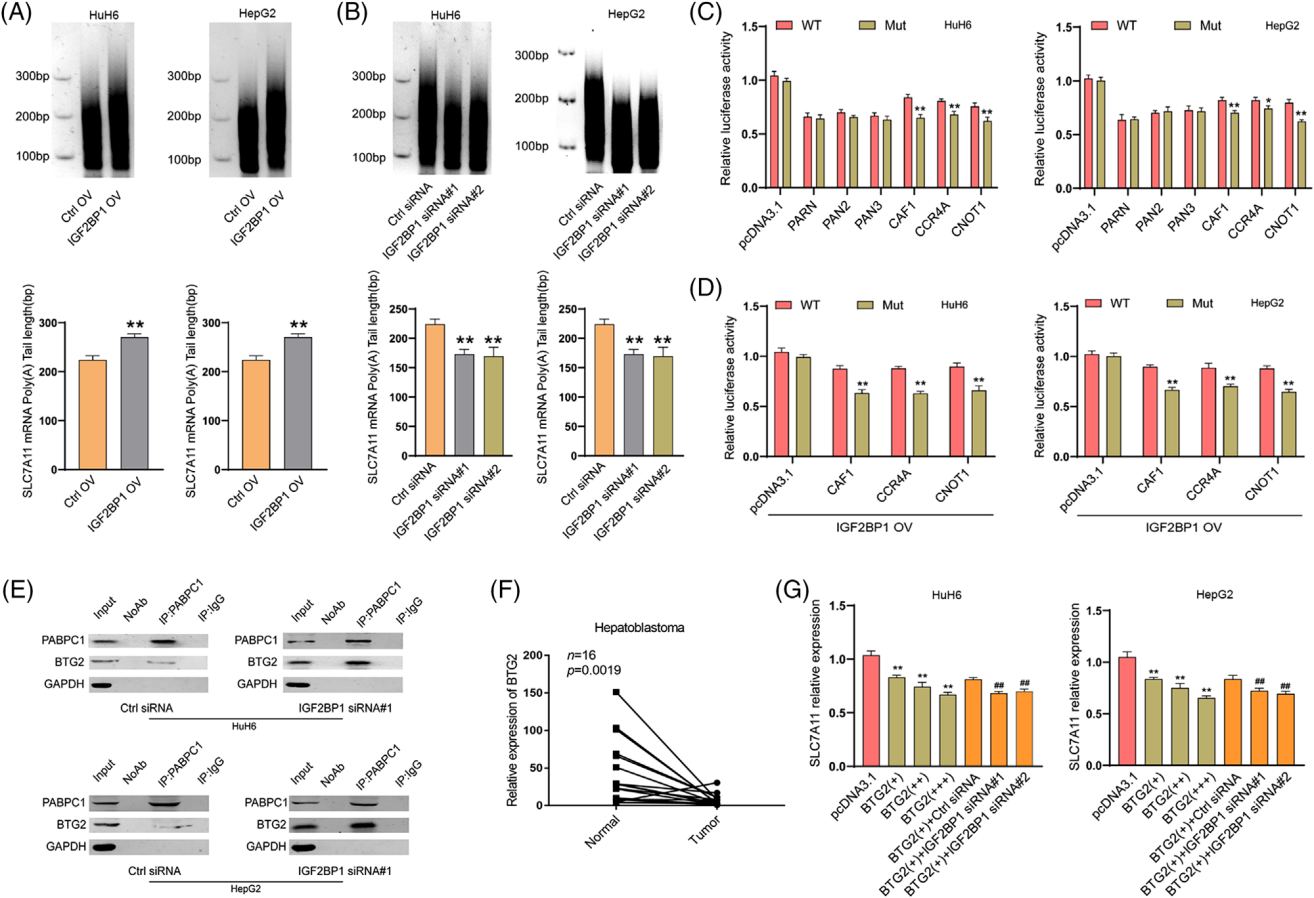
IGF2BP1 competitively binds to PABPC1 to inhibit the deadenylation of *SLC7A11* mRNA regulated by the BTG2/CCR4‐NOT complex. Poly(A) tail length of the endogenous *SLC7A11* transcript under IGF2BP1 overexpression (A) or deletion (B) was determined via the RACE‐PAT assay in HuH6 and HepG2 cells. (C) Dual luciferase reporter assay showed the increased relative luciferase activities of WT pmir‐GlO reporter upon CAF1, CCR4A, or CNOT1 overexpression compared to Mut pmir‐GlO reporter in HuH6 and HepG2 cells. (D) Overexpressing IGF2BP1 enhanced the relative luciferase activities of the WT pmir‐GlO reporter under CAF1, CCR4A, or CNOT1 overexpression compared to Mut pmir‐GlO reporter in HuH6 and HepG2 cells. (E) Co‐immunoprecipitation of PABPC1 with BTG2 in HuH6 and HepG2 cells under IGF2BP1 knockdown. F. Relative expression of BTG2 in human HB and matched normal tissues was determined via RT‐qPCR (*n* = 16). (G) The mRNA levels of *SLC7A11* under BTG2 gradient expression and IGF2BP1 deficiency were measured via RT‐qPCR in HuH6 and HepG2 cells. IP, immunoprecipitation; Ctrl, control; WT, wide type; Mut, mutation. All quantitative data are presented as the means ± SD of three independent experiments. *p < 0.05, **/##*p*  <  0.01

The 3′poly(A) tail of mRNAs is essential for eukaryotic gene expression. Shortening the poly(A) tail (deadenylation) was reported to repress expression by reducing mRNA stability.^25^ The deadenylation process is triggered by deadenylases, which include the PARN, PAN2‐PAN3 complex and CCR4‐NOT complex in mammals.^19^ To investigate whether the IGF2BP1‐mediated enhancement of *SLC7A11* mRNA stability and expression is related to deadenylation, a RACE‐PAT assay was performed to measure the *SLC7A11* mRNA poly(A) tail length. Knockdown of CNOT1, a large scaffold subunit of the CCR4‐NOT complex, significantly increased the length of the *SLC7A11* mRNA poly(A) tail in HuH6 cells, but this effect was not observed upon silencing PAN2, PAN3, or PARN (Figure S7A,B), suggesting that the CCR4‐NOT complex mediates *SLC7A11* mRNA deadenylation. Furthermore, we found that the length of the *SLC7A11* mRNA poly(A) tail was increased after IGF2BP1 overexpression and decreased after IGF2BP1 knockdown (Figure 4A,B; Figure S8A). These results demonstrated that IGF2BP1 negatively regulates the deadenylation of *SLC7A11* mRNA. We then performed a dual‐luciferase assay under PARN, PAN2, PAN3, CAF1, CCR4A, or CNOT1 overexpression in HuH6 and HepG2 cells (Figure S8A). We found that CAF1, CCR4A and CNOT1 overexpression accelerated the deadenylation of the reporter vector with Mut *SLC7A11* 3′UTR, whereas such acceleration was diminished for the WT reporter, suggesting that the m6A modification could protect *SLC7A11* mRNA from deadenylation (Figure 4C). Moreover, IGF2BP1 overexpression enhanced the protective effect of the m6A modification (Figure 4D). Taken together, these results implied that IGF2BP1 promoted *SLC7A11* mRNA stability and expression through suppression of its CCR4‐NOT‐regulated deadenylation in an m6A‐dependent manner.

### 3.7 IGF2BP1 competitively binds to PABPC1 to block it from recruiting the BTG2/CCR4‐NOT complex

IGF2BP1 was previously reported to bind to PABPC1 and enhance mRNA stability.^26^ The CCR4‐NOT complex can be recruited to PABPC1 by BTG2, which binds to the mRNA poly(A) tail, thereby stimulating mRNA deadenylation.^18^ To test whether IGF2BP1 enhanced the stability and expression of *SLC7A11* mRNA through binding PABPC1 to prevent the recruitment of BTG2/CCR4‐NOT, a Co‐IP assay was performed under treatment with an RNase inhibitor or RNase A. Western blotting analysis validated the interaction between IGF2BP1 and PABPC1 in HuH6 cells, and this interaction relied on the presence of RNA (Figure S9A,B). PABPC1 was co‐precipitated with BTG2, CNOT1, CAF1 and CCR4A, which is consistent with previous reports,^18^, ^26^ and our results also suggested that the interaction of PABPC1 with CNOT1, CAF1, as well as CCR4A might rely on the presence of RNA and BTG2 (Figure S9C,D). Next, we examined the interaction between PABPC1 and BTG2 after IGF2BP1 knockdown in HuH6 and HepG2 cells. Results from the Co‐IP assay showed that knocking down IGF2BP1 strengthened the PABPC1‐BTG2 interaction (Figure 4E). Thus, IGF2BP1 could compete with BTG2 to bind to PABPC1 and block the recruitment of the CCR4‐NOT complex to PABPC1. BTG2 is a member of the BTG/Tob protein family, which acts as a tumour suppressor by controlling mRNA stability. It is also a general activator of mRNA deadenylation.^16^ In HB tissues, the expression of BTG2 was significantly downregulated (Figure 4F), as was also observed for other solid tumours.^27^ To examine the inhibitory effect of BTG2 on SLC7A11, we determined the SLC7A11 expression under BTG2 overexpression. RT‐qPCR results showed that overexpression of BTG2 attenuated the expression of SLC7A11 in a concentration‐dependent manner. In addition, BTG2 overexpression followed by IGF2BP1 knockdown aggravated the inhibitory effect of BTG2 on the SLC7A11 expression (Figure 4G; Figure S8A), suggesting that IGF2BP1 inhibited the BTG2‐induced deadenylation of *SLC7A11* mRNA. To further confirm that the METTL3/IGF2BP1/m6A modification could prevent *SLC7A11* mRNA from deadenylation, we transfected the *SLC7A11* 3′UTR‐linked luciferase reporter plasmid into HuH6 cells. The RACE‐PAT assay demonstrated that IGF2BP1 deficiency diminished the inhibitory effect of BTG2 depletion on deadenylation in an m6A‐dependent manner (Figure S9E,F). Taken together, these results illustrated that IGF2BP1 enhances the stability and expression of *SLC7A11* mRNA by competitively binding PABPC1 to prevent BTG2/CCR4‐NOT complex recruitment, thereby suppressing the deadenylation of *SLC7A11* mRNA.

### 3.8 METTL3 enhances ferroptosis resistance in HB cells

**FIGURE 5:**
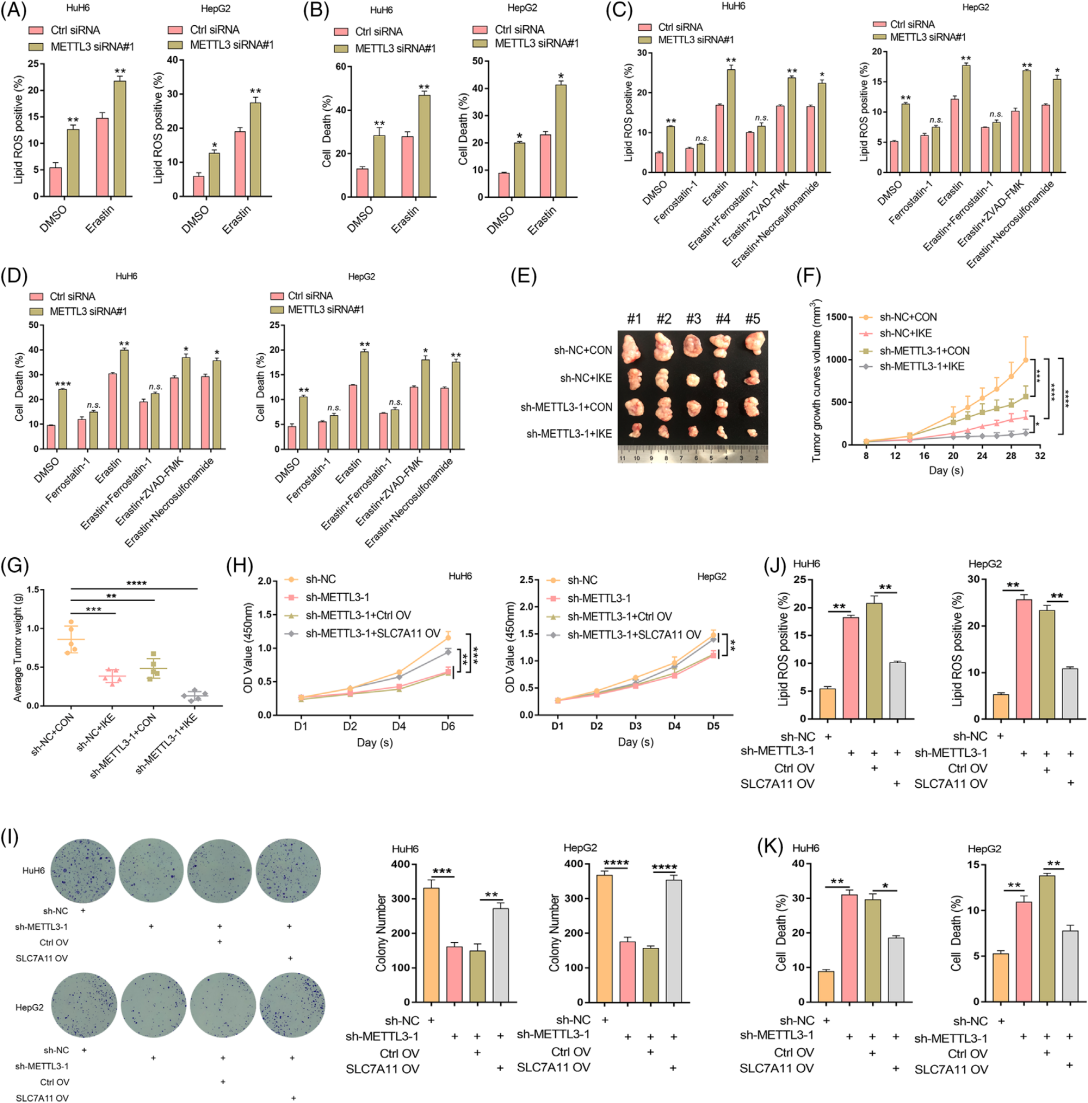
Knocking down METTL3 enhances the sensitivity of HB cells to ferroptosis. HuH6 and HepG2 cells transfected with METTL3 siRNA#1 were treated with erastin and lipid ROS levels (A) as well as cell death (B) were then measured via flow cytometry. HuH6 and HepG2 cells transfected with METTL3 siRNA#1 were treated with ferrostatin‐1, erastin, erastin + ferrostatin‐1, erastin + ZVAD‐FMK, or erastin + necrosulfonamide, respectively, and lipid ROS levels (C) as well as cell death (D) were then measured via flow cytometry. (E) Tumour images of the resected sh‐NC or sh‐METTL3‐1 tumours treated with or without IKE. (F) Tumour volumes were measured using an electronic calliper every 2 days and calculated using the formula volume (mm^3^) = Length (mm) × Width^2^ (mm^2^)/2. (G) Tumour weights of the resected sh‐NC or sh‐METTL3‐1 tumours treated with or without IKE. The proliferation of HuH6 and HepG2 cells stably transfected with sh‐METTL3 under SLC7A11 overexpression was assessed via CCK8 (H) and colony formation assays (I). The levels of lipid ROS (J) and cell death (K) in HuH6 and HepG2 cells stably transfected with sh‐METTL3 upon SLC7A11 overexpression were measured via flow cytometry. Erastin: HepG2 (30 μM) and HuH6 (20 μM), Ferrostatin‐1: 1 μM, ZVAD‐FMK: 10 μM, Necrosulfonamide: 1 μM, GSH: 0.8 mM. Ctrl, control; OV, overexpression vector. All quantitative data are presented as the means ± SD of three independent experiments. n.s., no significant difference, *p < .05, **p < .01, ***p < .001, ****p < .0001

Repressing SLC7A11 expression can result in decreased cystine uptake and increased ferroptosis sensitivity.^5^, ^7^ Therefore, we investigated whether the downregulation of SLC7A11 induced by METTL3 knockdown could enhance the sensitivity of HB cells to erastin. Our results showed that knockdown of METTL3 in both HuH6 and HepG2 cells significantly increased lipid ROS levels, MDA concentration, 4‐HNE and cell death induced by erastin (Figure 5A,B, Figure S10B,C), suggesting that METTL3 deletion enhanced the ferroptosis sensitivity of HB cells. Importantly, the increased lipid ROS levels and cell death induced by METTL3 deficiency could be rescued via treatment with ferrostatin‐1. Further, ferrostatin‐1 suppressed the synergistic effect of METTL3 deficiency and erastin as opposed to ZVAD‐FMK or necrosulfonamide (Figure 5C,D). In addition, the GSH/GSSG ratio dramatically decreased upon METTL3 knockdown, and the METTL3 deficiency‐induced increase in lipid ROS levels and cell death could be rescued via GSH supplementation (Figure S10A,D,E). To further confirm the regulatory effect of METTL3 on ferroptosis sensitivity, ten nude mice (five in each of the IKE and vehicle groups) were injected subcutaneously with sh‐METTL3 HuH6 cells or sh‐NC HuH6 cells. METTL3 knockdown dramatically enhanced the inhibitory effect of IKE on HB cell growth in vivo (Figure 5E–G). Moreover, METTL3 deletion aggravated the IKE‐induced increase in MDA concentration and decrease in GSH/GSSG ratio in subcutaneous xenograft tumours (Figure S10F,G). Taken together, these results convincingly demonstrated that the METTL3‐catalyzed m6A methylation of *SLC7A11* mRNA can promote HB cell ferroptosis resistance.

### 3.9 SLC7A11 overexpression rescues METTL3 knockdown‐mediated ferroptosis

To further confirm the enhancing effect of m6A methylation‐mediated SLC7A11 upregulation on HB ferroptosis resistance, we re‐expressed SLC7A11 in sh‐METTL3 HepG2 and HuH6 cells and subjected them to CCK8, colony formation and flow cytometry assays. These results showed that re‐expression of SLC7A11 rescued the inhibitory effect of METTL3 deletion on HB cell proliferation (Figure 5H,I). Moreover, the increased lipid ROS levels and HB cell death caused by METTL3 knockdown were also rescued by re‐expressing SLC7A11 (Figure 5J,K). Furthermore, 4‐HNE levels and the GSH/GSSG ratio confirmed the regulatory role of METTL3‐mediated m6A modification of SLC7A11 in HB cell ferroptosis (Figure S10H,I). Collectively, these results supported the notion that m6A methylation upregulates SLC7A11, thereby enhancing HB ferroptosis resistance and promoting HB tumorigenesis.

## 4 DISCUSSION

Ferroptosis is a novel form of regulated cell death that results from the accumulation of iron‐dependent lipid peroxide.^29^ It plays a central role in regulating the growth and proliferation of various cancer cell types, including colorectal cancer, hepatocellular carcinoma, gastric cancer and ovarian cancer cells.^30^ Moreover, ferroptosis is recognized as a mechanism for the eradication of malignant cells, and accumulating evidence has elucidated the potential of triggering ferroptosis for cancer therapy.^29^, ^30^ System Xc^−^, a sodium‐independent cysteine/glutamate antiporter, is able to maintain redox homeostasis by importing cystine, which is then converted to cysteine for synthesizing the antioxidant GSH. Inhibiting system Xc^−^ activity can trigger ferroptosis.^31^ The antiporter is composed of heavy chain subunit SLC3A2 (CD98hc) and light chain subunit SLC7A11 (xCT).^32^ SLC7A11, as the catalytic subunit of the Xc^−^ system, is overexpressed in multiple human tumours. Further, suppressing SLC7A11 expression can result in lipid peroxidation and ferroptosis, thereby promoting tumour cell death.^8^, ^33^ However, the role and molecular mechanisms of SLC7A11‐guided ferroptosis in HB progression remain unknown. Thus, we aimed to investigate the function of SLC7A11 in HB tumorigenesis. Our results indicated that SLC7A11 is highly expressed in HB tissues and cell lines, and its downregulation could suppress cell proliferation in vitro and in vivo. Moreover, inhibiting SLC7A11 can enhance the sensitivity of HB cells to ferroptosis inducer erastin. These results demonstrated that SLC7A11 acts as an oncogene that promotes HB proliferation and enhances tumour cell ferroptosis resistance.

It has been established that *SLC7A11* expression can be regulated by transcription factors and epigenetic mechanisms at the transcriptional level as well as by post‐transcriptional regulatory mechanisms.^8^ The present results indicated that SLC7A11 expression is regulated via epigenetic modifications in HB tissues and cell lines. The m6A modification of RNA is among the most important mechanisms of epigenetic regulation. Our previous work demonstrated that this modification is abundant in HB tissues, with METTL3 being the main factor responsible for aberrant m6A modification.^15^ The m6A modification has been reported to participate in almost all steps of RNA metabolism, including miRNA processing, mRNA degradation and protein translation, and so forth.^12^, ^34^ Herein, we verified that *SLC7A11* mRNA undergoes abnormal METTL3‐mediated m6A modification, which can enhance its stability and expression. The m6A modification is recognized and bound by specific m6A reader proteins.^34^ To date, IGF2BP1‐3, YTHDF2, YTHDF3 and YTHDC2 have been reported to act as readers and regulate mRNA stability.^24^ In this study, we found that IGF2BP1 can recognize and bind to the m6A modification in *SLC7A11* mRNA, stabilizing it and upregulating its expression in an m6A‐dependent manner. Furthermore, IGF2BP1 expression was upregulated in HB, consistent with its role in promoting SLC7A11 expression.

**FIGURE 6:**
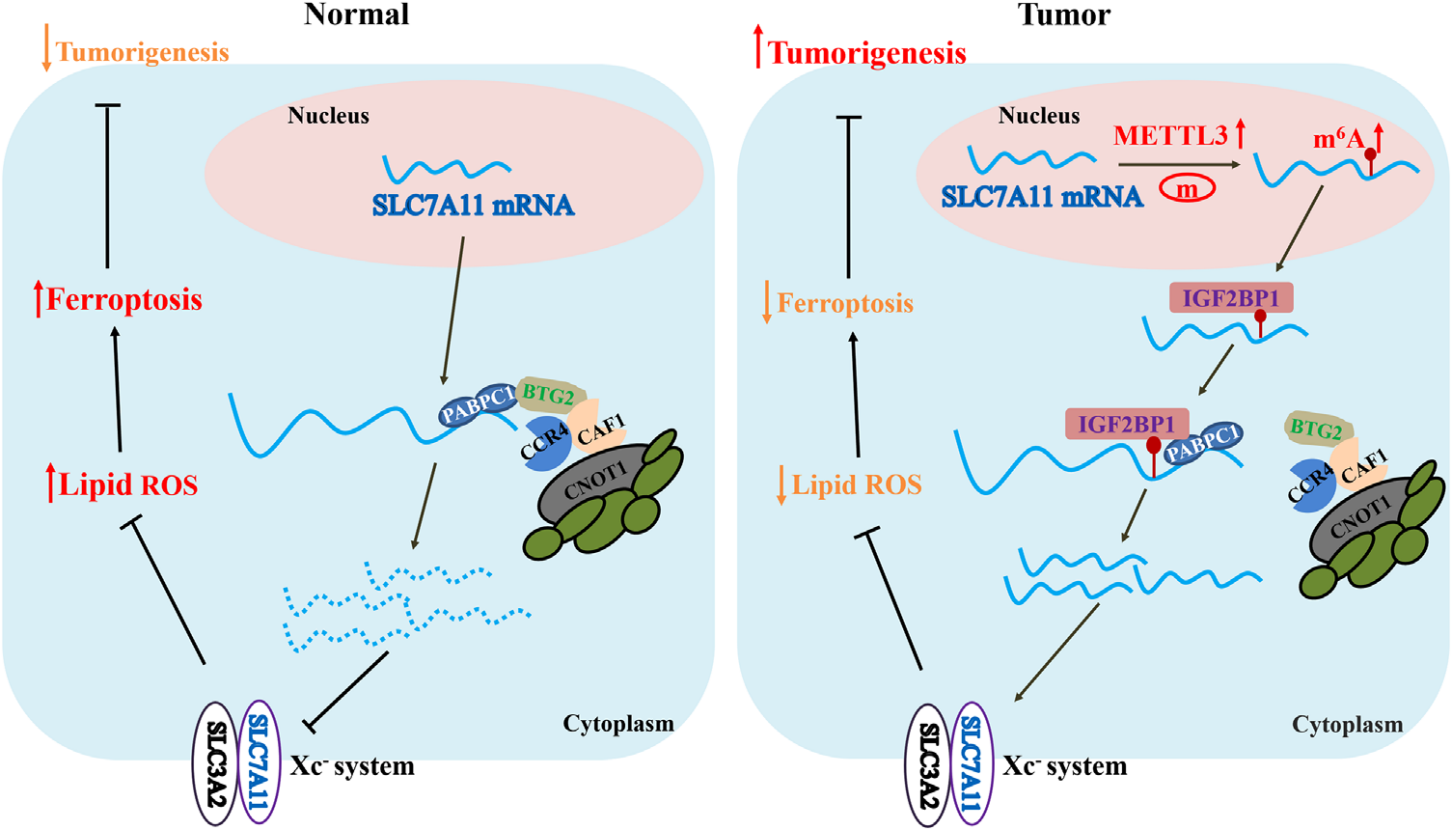
A diagram illustrating the underlying molecular mechanism identified in this study. The diagram depicts the m6A‐dependent regulation of *SLC7A11* mRNA that promotes the ferroptosis resistance of HB cells. Mechanistically, IGF2BP1 recognizes the METTL3‐mediated m6A modification of *SLC7A11* mRNA and sustains its stability and expression through competitively binding to PABPC1. The interaction between IGF2BP1 and PABPC1 blocks recruitment of the BTG2/CCR4‐NOT complex to PABPC1 and inhibits the deadenylation of *SLC7A11* mRNA

In eukaryotes, poly(A) tails play a central role in the translation and stabilization of mRNAs. Removing the poly(A) tail (deadenylation) is the rate‐limiting step in mRNA degradation, which can repress gene expression by decreasing the mRNAs stability.^35^, ^36^, ^37^ Three deadenylase complexes contribute to deadenylation activity in mammals, including the PARN, CCR4‐NOT complex and PAN2‐PAN3 complex, among which CCR4‐NOT complex is considered to play the major role. The CCR4‐NOT complex is composed of seven core subunits and two poly(A)‐selective deadenylases, CCR4/CCR4A and CAF1/POP2.^19^, ^25^ Recent studies have revealed that m6A‐containing mRNAs exhibit accelerated deadenylation mediated via the CCR4‐NOT complex that is directly recruited by YTHDF2.^19^, ^38^ Our results demonstrated that the CCR4‐NOT complex also mediates *SLC7A11* mRNA deadenylation, yet this process can be antagonized by IGF2BP1. Moreover, IGF2BP1 inhibited *SLC7A11* mRNA deadenylation regulated by the CCR4‐NOT complex in an m6A‐dependent manner. YTHDF2 was reported to destabilize m6A‐containing RNAs by directly recruiting the CCR4‐NOT complex.^19^ However, the underlying mechanism through which IGF2BP1 enhances *SLC7A11* mRNA stability by suppressing CCR4‐NOT function remains unclear. IGF2BP1 was reported to enhance mRNA stability by interacting with stabilizers, such as PABPC1.^26^, ^39^ PABPC1 is a poly(A)‐binding protein that can promote mRNA deadenylation by recruiting BTG2 to bridge PABPC1 RNA‐binding domains and CAF1 deadenylase.^17^, ^18^ Our results confirmed the interaction of IGF2BP1 with PABPC1, in addition to those between PABPC1 and BTG2, CNOT1, CAF1, as well as CCR4A. The interaction between IGF2BP1 and PABPC1 is dependent on RNA. Meanwhile, the interactions between PABPC1 and CNOT1/CAF1/CCR4A are dependent on both RNA and BTG2. Furthermore, our results demonstrated that IGF2BP1 competitively binds to PABPC1, thereby blocking the recruitment of the BTG2/CCR4‐NOT complex. BTG2 is a tumour suppressor that is downregulated in some cancer cell types.^27^ In this study, we found that BTG2 decreased the expression of SLC7A11 in a level‐dependent manner and that knocking down IGF2BP1 aggravated the inhibition of SLC7A11 expression. Moreover, suppression of BTG2 expression upregulated SLC7A11, and IGF2BP1 deficiency diminished this effect in an m6A‐dependent manner. Taken together, these results indicated that IGF2BP1 can inhibit the deadenylation of *SLC7A11* mRNA through competitively binding to PABPC1 and blocking recruitment of the BTG2/CCR4‐NOT complex to PABPC1, with this inhibitory effect of IGF2BP1 being m6A‐dependent (Figure 6).

Previous studies have demonstrated that transcriptional and post‐transcriptional suppression of SLC7A11 by transcription factors (such as ATF3 and ATF4), H2A deubiquitinases (such as BAP1), and epigenetic modifications (such as H2Bub1) can promote erastin‐induced tumour cell ferroptosis.^40^, ^41^, ^42^, ^43^ Consistently, our results showed that the downregulation of m6A modifications under METTL3 deletion inhibited SLC7A11 expression and increased the levels of lipid ROS and cell ferroptosis. Moreover, METTL3 deficiency enhanced the sensitivity of HB cells to ferroptosis (erastin treatment in this study) both in vitro and in vivo. Taken together, the current findings highlight the m6A modification of SLC7A11 as a potential therapeutic target in HB.

In conclusion, this study elucidated the oncogenic role of *SLC7A11* in HB tumorigenesis exerted via promotion of ferroptosis resistance. The METTL3/IGF2BP1/m6A modification enhances the stability and expression of *SLC7A11* mRNA thus facilitating SLC7A11‐guided ferroptosis. In addition, METTL3 deficiency enhanced the sensitivity of HB cells to ferroptosis, suggesting that blockade of the m6A‐SLC7A11 axis might represent a potential therapeutic approach against HB.

## CONFLICT OF INTEREST

All authors declare that there is no conflict of interest.

## Supporting information

Supporting information.

Supporting information.

Supporting information.

Supporting information.

Supporting information.

Supporting information.

Supporting information.

Supporting information.

Supporting information.

Supporting information.

Supporting information.

Supporting information.

Supporting information.
figure‐legends

Supporting information.

Supporting information.

Supporting information.
