# Supplementary material for: The N6‐methyladenosine modification enhances ferroptosis resistance through inhibiting SLC7A11 mRNA deadenylation in hepatoblastoma
Source: Clin Transl Med. 2022 May 6;12(5):e778. doi: 10.1002/ctm2.778 (PMC9076012; doi:10.1002/ctm2.778)
Supplement: Supplementary file 1 — Supporting information. [file CTM2-12-e778-s008.docx]

1. Putative m6A sites within SLC7A11 3’UTR：


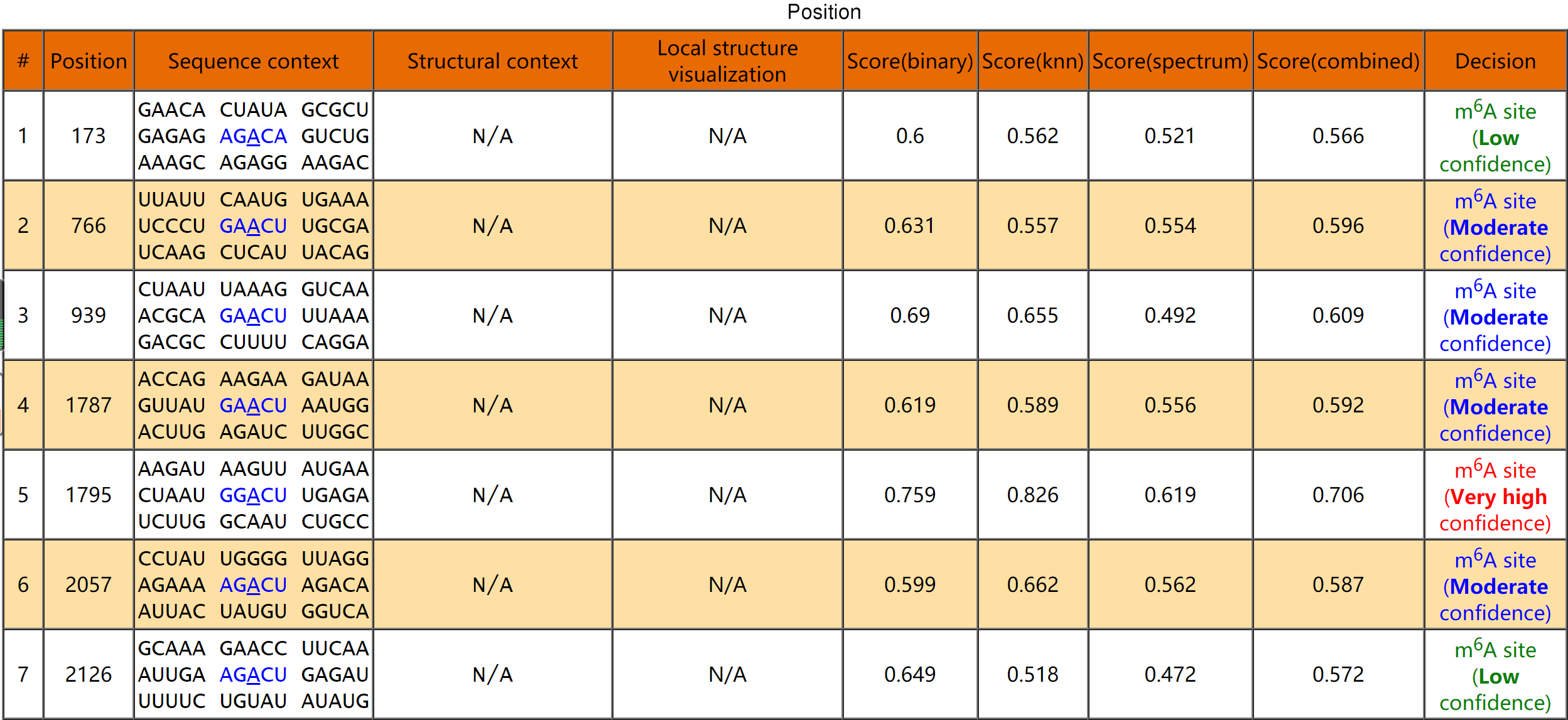


1. The sequences were inserted:

WT:

actaatggacttgagatcttggcaatctgcccaaggggagacacaaaatagggatttttacttcattttctgaaagtctagagaattacaactttggtgataaacaaaaggagtcagttatttttattcatatattttagcatattcgaactaatttctaagaaatttagttataactctatgtagttatagaaagtgaatatgcagttattctatgagtcgcacaattcttgagtctctgatacctacctattggggttaggagaaaagactagacaattactatgtggtcattctctacaacatatgttagcacggcaaagaaccttcaaattgaagactgagatttttctgtatatatgggttttgtaaagatggttttacacactatagatgtctatactgtgaaaagtgttttcaattctgaaaaaaagcatacatcatgattatggcaaagaggagagaaagaaatttattttacattgacattgcattgcttc

Mut:

actaatggCcttgagatcttggcaatctgcccaaggggagacacaaaatagggatttttacttcattttctgaaagtctagagaattacaactttggtgataaacaaaaggagtcagttatttttattcatatattttagcatattcgaactaatttctaagaaatttagttataactctatgtagttatagaaagtgaatatgcagttattctatgagtcgcacaattcttgagtctctgatacctacctattggggttaggagaaaagactagacaattactatgtggtcattctctacaacatatgttagcacggcaaagaaccttcaaattgaagactgagatttttctgtatatatgggttttgtaaagatggttttacacactatagatgtctatactgtgaaaagtgttttcaattctgaaaaaaagcatacatcatgattatggcaaagaggagagaaagaaatttattttacattgacattgcattgcttc

1. SLC7A11 3’UTR Probes:

A probe: ACUAAUGGACUUGAGAUCUUGGCAAUCUGCCCAAGGGGAGACACAAAAUA

m6A probe: ACUAAUGGm6ACUUGAGAUCUUGGCAAUCUGCCCAAGGGGAGACACAAAAUA

UTR-NC: GGGAUUUUUACUUCAUUUUCUGAAAGUCUAGAGAAUUACAACUUUGGUGA
