# Supplementary material for: The N6‐methyladenosine modification enhances ferroptosis resistance through inhibiting SLC7A11 mRNA deadenylation in hepatoblastoma
Source: Clin Transl Med. 2022 May 6;12(5):e778. doi: 10.1002/ctm2.778 (PMC9076012; doi:10.1002/ctm2.778)
Supplement: Supplementary file 2 — Supporting information. [file CTM2-12-e778-s014.docx]

**Materials and methods**

**mRNA sequencing (mRNA-seq)**

Total RNA was extracted from five human HB tissues and five paired normal tissues using TRIzol reagent (Invitrogen) with DNase treatment. The rRNAs were then removed using the Ribo-Zero rRNA Removal Kit (Illumina), and the RNA libraries were constructed using TruSeq Stranded Total RNA Library Prep Kit (Illumina). Quality-controlled RNA libraries were quantified using the Bioanalyzer 2100 system (Agilent Technologies) and then sequenced on an Illumina HiSeq 4000 sequencer.

**Cell transfection and lentivirus transduction**

When the confluence of HepG2 or HuH6 cells reached about 30%, the cells were transfected with chemically synthesized small interfering RNAs (siRNAs). These siRNAs against target genes were purchased from GenePharma (Shanghai, China). Overexpression vector was established through amplifying the coding sequences (CDSs) of the target genes and subcloning the CDSs into pcDNA 3.1 vector (Invitrogen), with an empty vector as the negative control. Plasmids or oligonucleotides were transfected using the Lipofectamine 2000 reagent (Invitrogen) following the manufacturer’s instructions.

To construct stable knockdown cell lines, shRNA constructs against target genes and the control shRNA construct were obtained from GenePharma (Shanghai, China). Lentiviruses were packaged in HEK293T cells through co-transfecting each of the shRNA constructs with the packing vectors (PsPAX2, pMD2.G) into HEK293T cells. HepG2 and Huh6 cells were directedly infected with the lentivirus harvested at 24 h, 48 h, and 72 h under polybrene (Santa Cruz Biotechnology) treatment for 12-24 h. Then, positively transfected HepG2 and Huh6 cells were screened with 2 μg/mL puromycin (Invitrogen) treatment for 7-10 days. The sequences for siRNA and shRNA are listed in Table S1.

**RNA isolation and quantitative real-time PCR (RT-qPCR) assays**

Total RNA was extracted from tissues or treated cells using the TRIzol reagent (Invitrogen) according to manufacturer’s instructions. Reverse transcription was performed to synthesize cDNA using the PrimeScriptTM RT reagent Kit (TaKaRa). RT-qPCR was performed using KAPA SYBR® FAST qPCR Kit Master Mix (2X) Universal (Applied Biosystems) on the ABI 7500 real-time PCR System (Applied Biosystems). GAPDH was used to normalize the expression levels of target genes. The 2^-ΔΔCt^ method was employed to calculate the relative expression of target genes. The primers used are listed in Table S1.

**Western blotting assays and co-immunoprecipitation (Co-IP)**

Total proteins were isolated from tissues or treated cells using RIPA Lysis buffer (Beyotime) supplemented with phosphatase and protease inhibitors on ice for 30 min. The supernatant of the tissue or cell lysates was collected after centrifuged for 15 min at 4°C (12000 g). The protein concentration was quantified using a BCA protein assay kit (Thermo Scientific). After boiled in 6×loading buffer (Beyotime), the proteins were separated by SDS-PAGE and transferred onto a nitrocellulose membrane (GE Healthcare). The membrane was blocked with 5% nonfat milk (Mengniu) in PBST for 1 h, and was then incubated with primary antibodies overnight at 4°C. The membrane was washed with PBST for 3 times prior to incubation with goat anti-rabbit or goat anti-mouse secondary antibodies at room temperature. Finally, the chemiluminescent signal was detected via an ECL method.

Co-IP assay was performed in HB cells using the Dynabeads™ Co-Immunoprecipitation Kit (Thermo Scientific). According to the manufacturer’s instructions, cells were lysed with IP Lysis Buffer and mixed with the beads conjugated with indicated antibodies. Then, the immunoprecipitated protein complex was separated through boiling, followed by western blotting assays. All antibodies used in this study are listed in Table S2.

**CCK8 assays and colony formation assays**

Cell Counting Kit-8 (CCK8, Beyotime) was used to evaluate the cell proliferation ability. Briefly, treated cells were seeded in a 96-well plate in triplicate at a density of 1000 cells/200 μL per well. Then, the medium in each well was replaced with fresh medium (100 μL) containing 10 μL CCK8 reagent at indicated time points. Three hours later, the absorbance at 450 nm was determined by a multiplate reader (Bio Tek, Vermont, USA).

For colony formation assay, treated cells were plated in a 12-well plate at a density of 1000 cells per well. After being incubated for approximately 7 days, the cells were fixed with 4% paraformaldehyde (PFA) and stained with 1% crystal violet solution to visualize their colony-forming ability.

**RNA pulldown assays**

For RNA pulldown assay, SLC7A11 3’UTR probes with adenine (A) or m6A modification at the GGAC motif were synthesized (The sequences see in Data S1) and labeled with biotin. Then, proteins, isolated from HuH6 cells, were incubated with 3 μg biotinylated SLC7A11 3’UTR probes overnight at 4°C. Next, the biotin-conjugated RNA-protein complex was incubated with streptavidin magnetic beads (Life Technologies) for 4 h at 4°C. The streptavidin beads were boiled and subjected to western blotting assays.

**RNA immunoprecipitation (RIP)**

RIP assay was carried out using the Magna RIP Kit (Millipore) following the manufacturer’s instructions. Briefly, sufficient cell lysates were incubated with magnetic beads coupled with an antibody (5 μg) against IGF2BP1 (Abcam) or IgG (Millipore) at 4°C overnight. Then, the immunoprecipitated RNA-protein complex was treated with proteinase K to hydrolyze proteins, and the RNA was purified using TRIzol reagent (Invitrogen) for subsequent RT-qPCR detection. The primers used for RIP-qPCR are listed in Table S1.

**M6A immunoprecipitation and RT-qPCR (MeRIP-qPCR)**

Total RNA was isolated from treated cells using TRIzol reagent (Invitrogen). MeRIP-qPCR was performed as described in our previous work.^15^ Putative m6A sites within the 3’UTR of SLC7A11 mRNA were predicted using the SRAMP (<http://www.cuilab.cn/sramp>) and are highlighted in red color (See in Data S1). Primers were designed to cover these sites, with a limited product length of 100 nt. The MeRIP-qPCR primers used are listed in Table S1.

**Rapid amplification of cDNA ends-poly(A) test (****RACE-PAT)**

The length of the poly(A) tail of SLC7A11 mRNA was measured using RACE-PAT. Briefly, total RNA was reverse-transcribed with an oligo(dT) primer linked to an oligo(dT) anchor (See Table S1 for their sequences). Next, PCR amplification was performed with the SLC7A11 forward RACE primer and the oligo(dT)-anchor PCR primer, yielding a mixture of PCR-amplified products representing the length of the SLC7A11 poly(A) tail. PCR products were resolved on a 1.5% agarose gel.

**Dual-luciferase reporter assays**

The 3’UTR of SLC7A11 mRNA with wild-type m6A modification site (WT) or mutant m6A modification site (Mut, GGAC to GGCC) was cloned downstream of the Firefly luciferase-encoding region of the dual-luciferase reporter vector pmir-GLO (Promega) to construct the pmir-GLO-SLC7A11-3’UTR WT or pmir-GLO-SLC7A11-3’UTR Mut plasmid, respectively. HB cells with the indicated gene knockdown or overexpression were seeded in a 24-well plate. 24 h later, 0.8 μg of pmir-GLO-SLC7A11-3’UTR WT, pmir-GLO-SLC7A11-3’UTR Mut, or pmir-GLO plasmid was transfected into the cells using Lipofectamine 2000 (Invitrogen). Dual-luciferase reporter assay kit (Promega) was used to detect the Firefly and Renilla luciferase signals 24 h later. The sequences inserted into the pmir-GLO vector are shown in Data S1.

**Animal experiments**

Animal studies were approved by the Institutional Animal Care and Use Committee of Shanghai Children’s Medical Center. Four-week-old male nude mice (n = 24), obtained from Shanghai Super-B&K Laboratory Animal Crop (Shanghai, China), were subcutaneously injected with 1×10^7^ HuH6 cells infected with lentiviruses expressing shRNA against SLC7A11 (sh-SLC7A11) or its negative control (sh-NC) in the right flank. The tumors were measured every 2 days and their volume were calculated using the formula: Volume (mm^3^) = Length (mm)×Width^2^ (mm^2^)/2. Mice were then euthanized after 26 days and the tumor weight was measured.

Similarly, 20 male mice aged four weeks were subcutaneously injected with 1×10^7^ HuH6 cells infected with lentiviruses expressing shRNA against SLC7A11/METTL3 (sh-SLC7A11/METTL3) or its negative control (sh-NC) in the right flank. The tumor volume was measured using electronic caliper every 2 days. When the mean volume of tumors reached 90 mm^3^, mice were randomized into 4 groups and treated with a solvent composed of 65% D5W (5% dextrose in water), 5% Tween-80, and 30% PEG-400 or 50 mg/kg IKE, respectively, via intraperitoneal injection every other day. At the end of the study, Mice were euthanized and the tumor weight was measured.
