# Supplementary figures and images for: The N6‐methyladenosine modification enhances ferroptosis resistance through inhibiting SLC7A11 mRNA deadenylation in hepatoblastoma
Source: Clin Transl Med. 2022 May 6;12(5):e778. doi: 10.1002/ctm2.778 (PMC9076012; doi:10.1002/ctm2.778)

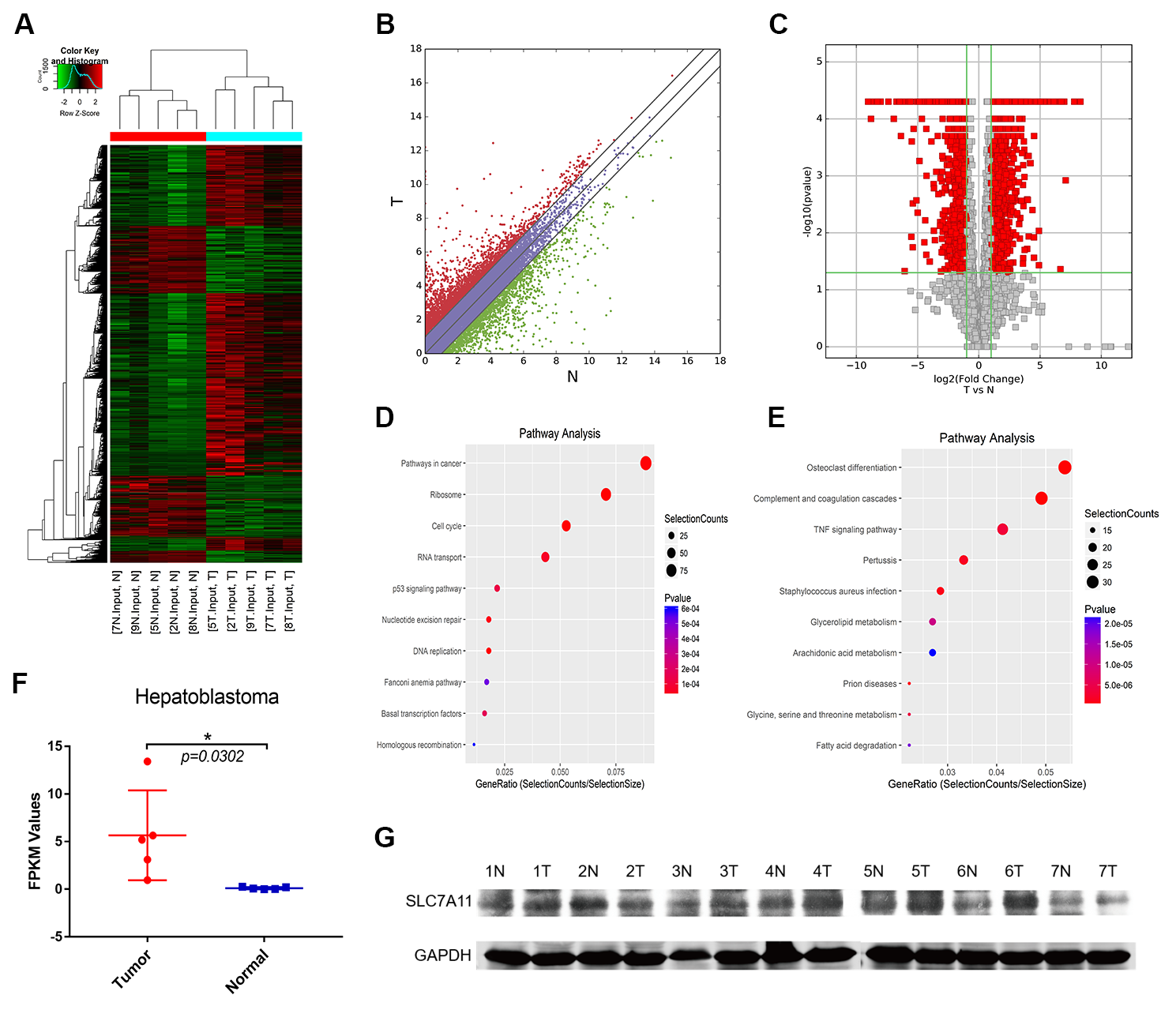

Supplement: Supplementary file 3 — Supporting information. [file CTM2-12-e778-s007.tif]

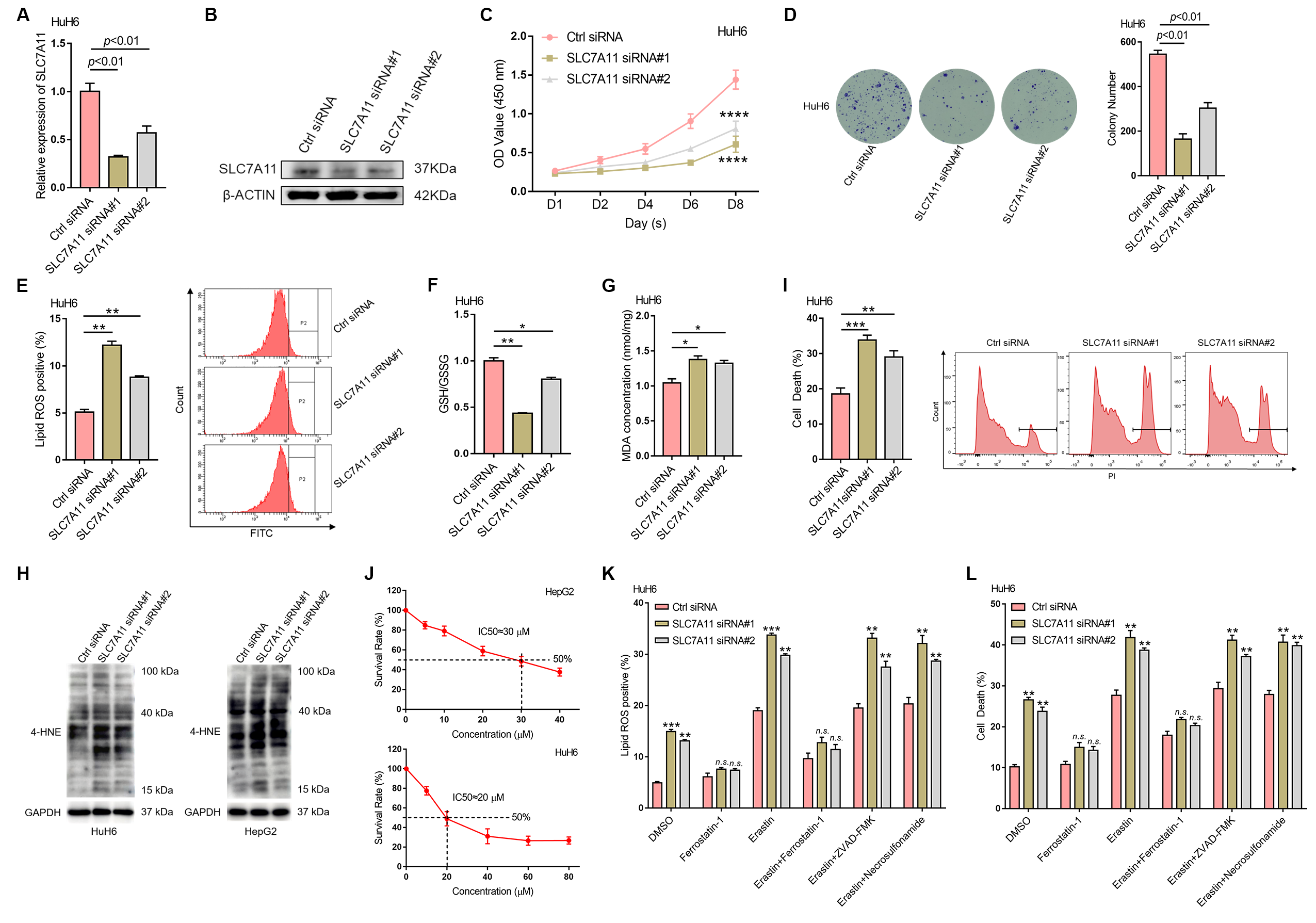

Supplement: Supplementary file 4 — Supporting information. [file CTM2-12-e778-s003.tif]

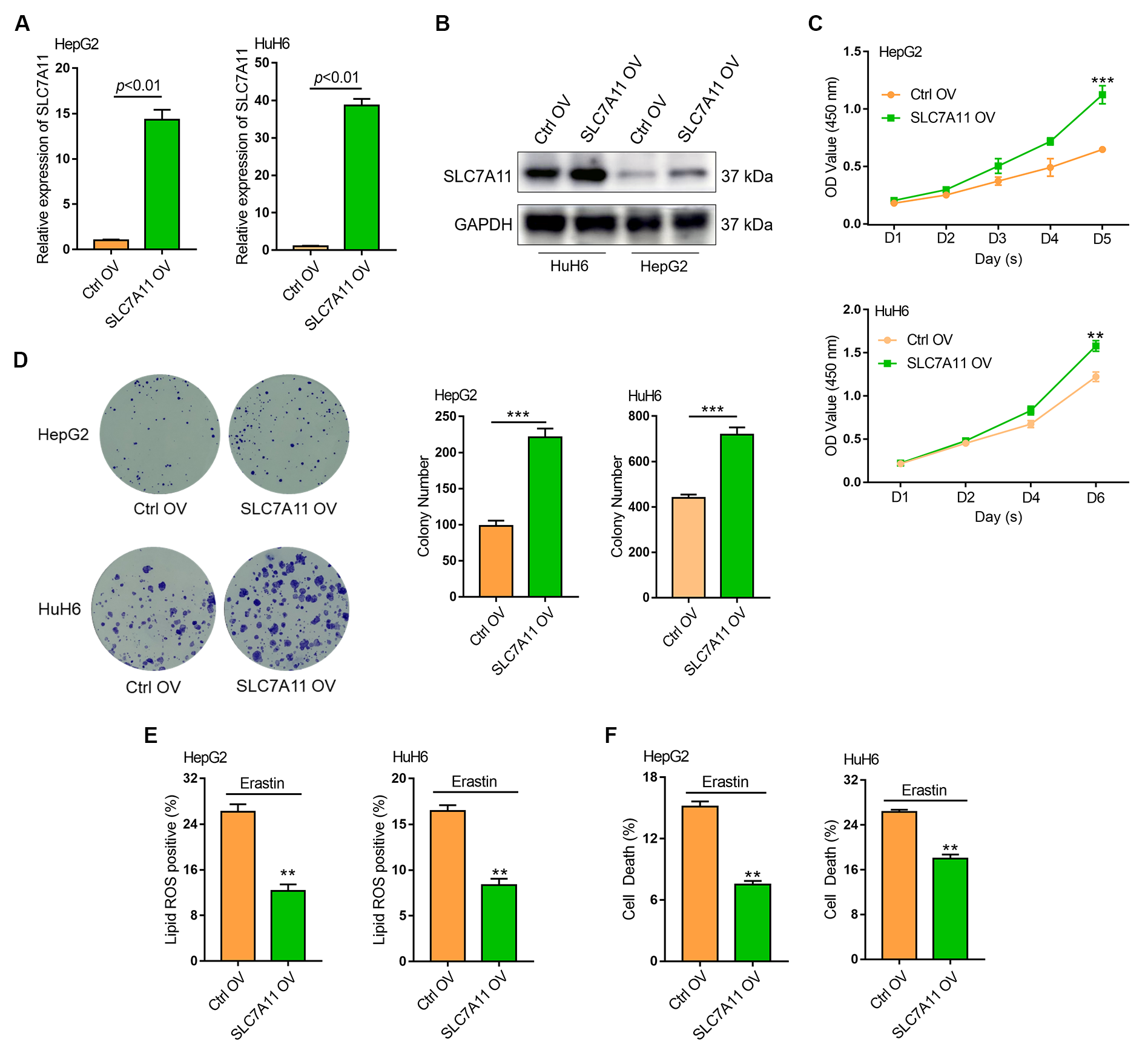

Supplement: Supplementary file 5 — Supporting information. [file CTM2-12-e778-s012.tif]

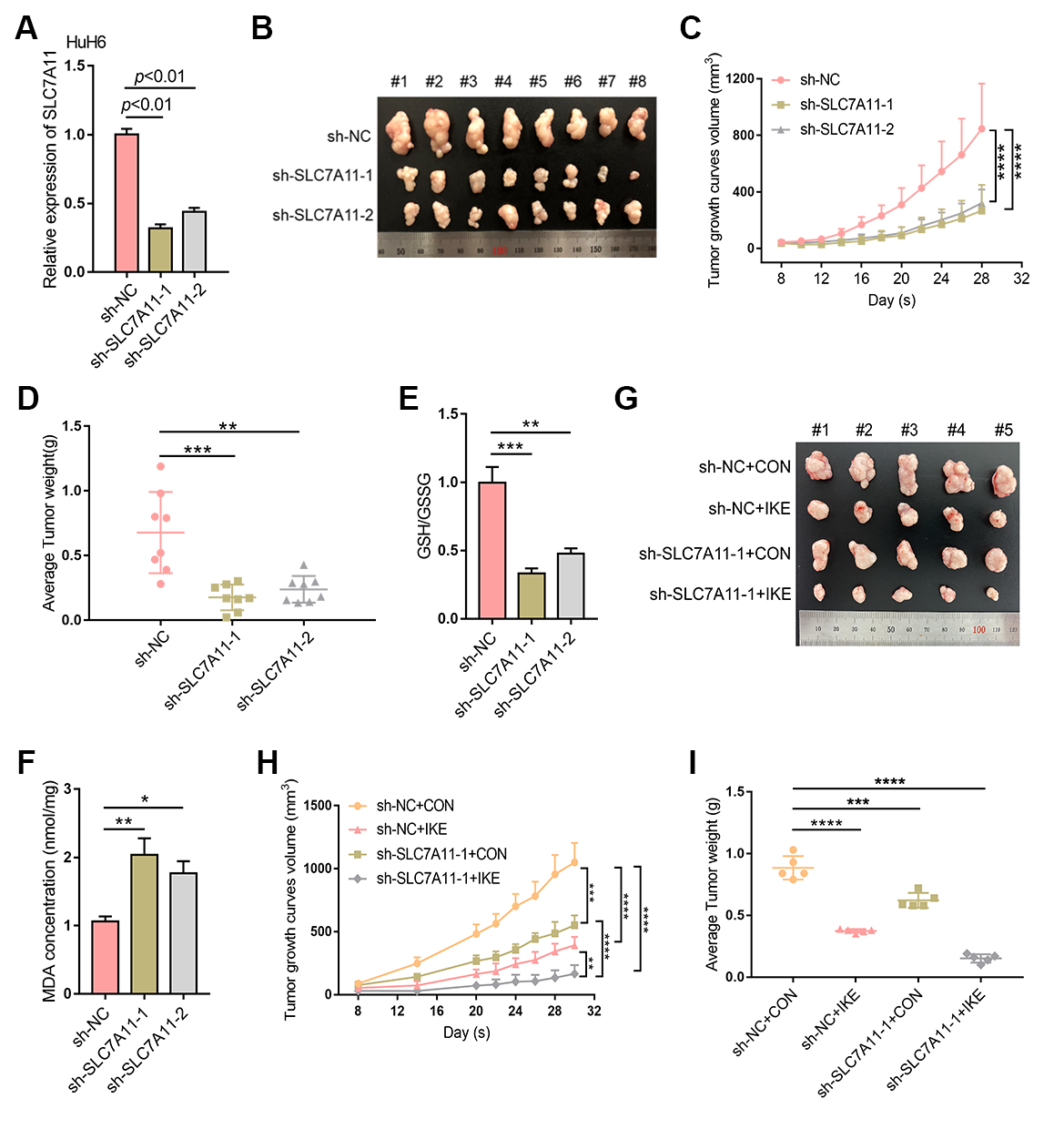

Supplement: Supplementary file 6 — Supporting information. [file CTM2-12-e778-s011.tif]

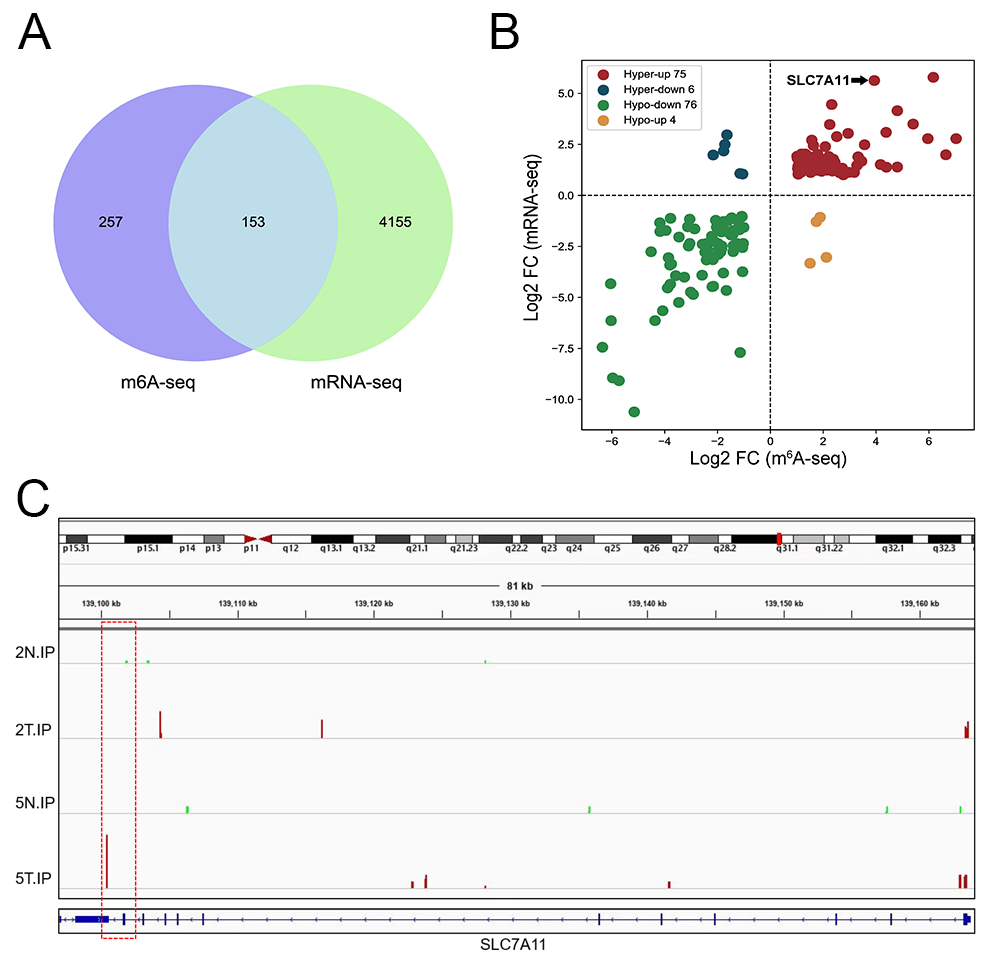

Supplement: Supplementary file 7 — Supporting information. [file CTM2-12-e778-s001.tif]

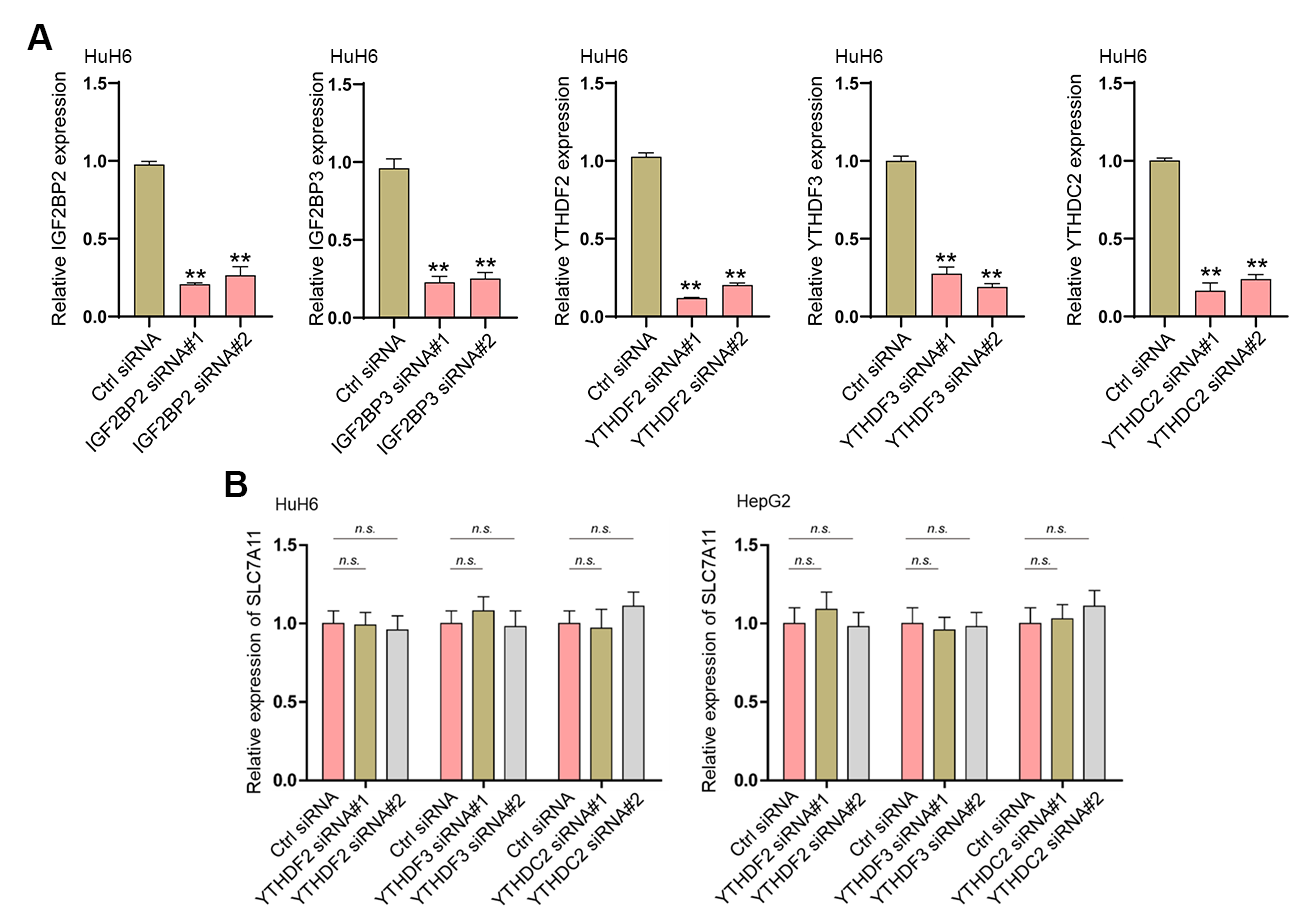

Supplement: Supplementary file 8 — Supporting information. [file CTM2-12-e778-s013.tif]

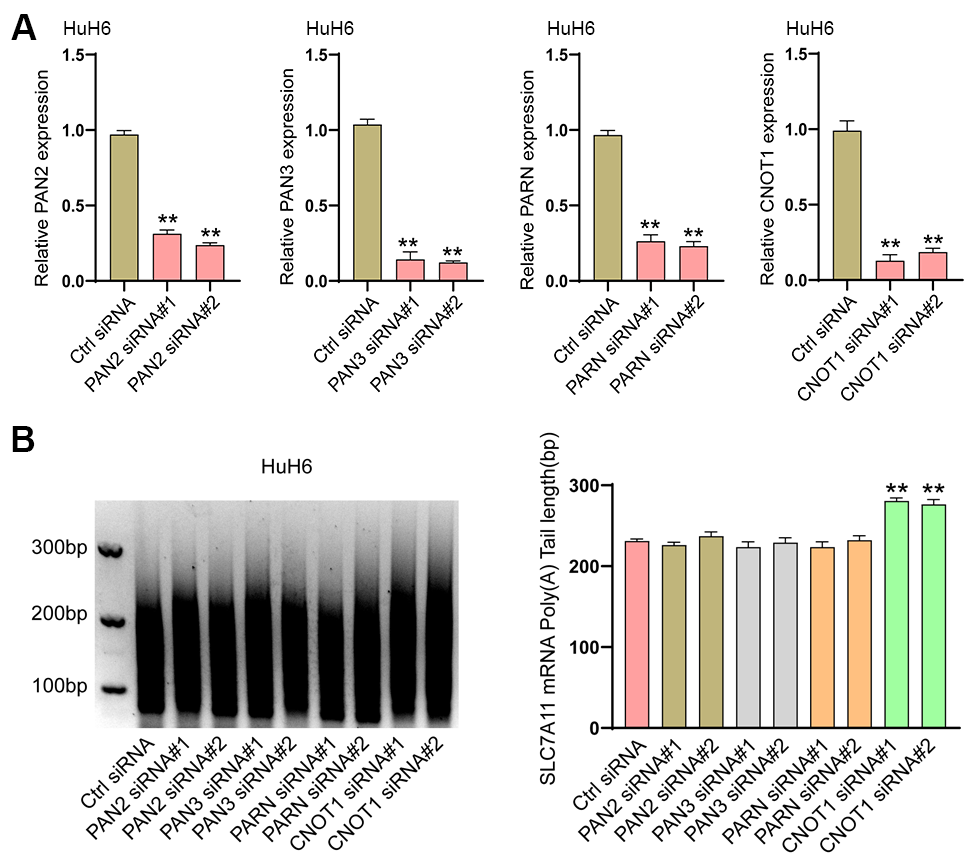

Supplement: Supplementary file 9 — Supporting information. [file CTM2-12-e778-s002.tif]

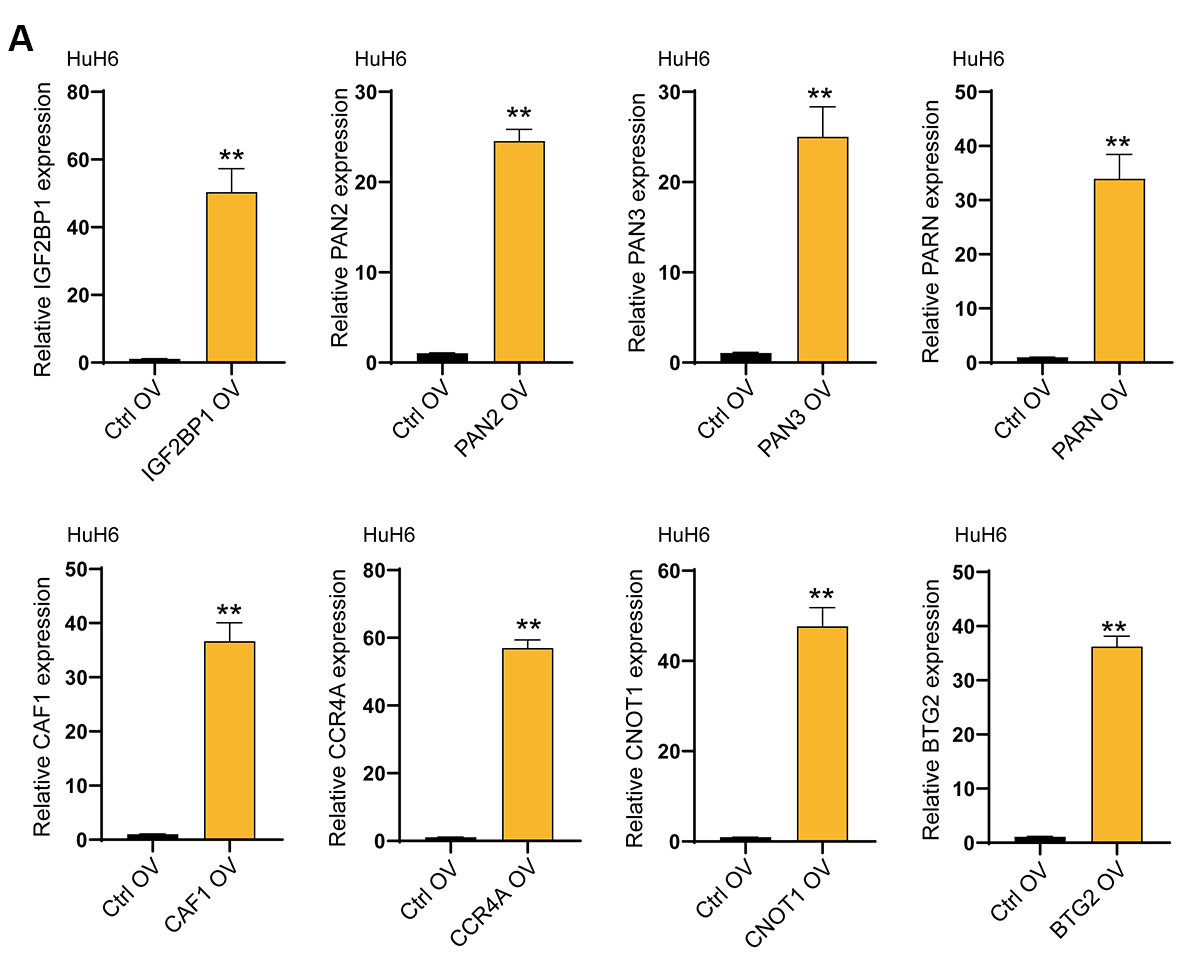

Supplement: Supplementary file 10 — Supporting information. [file CTM2-12-e778-s006.tif]

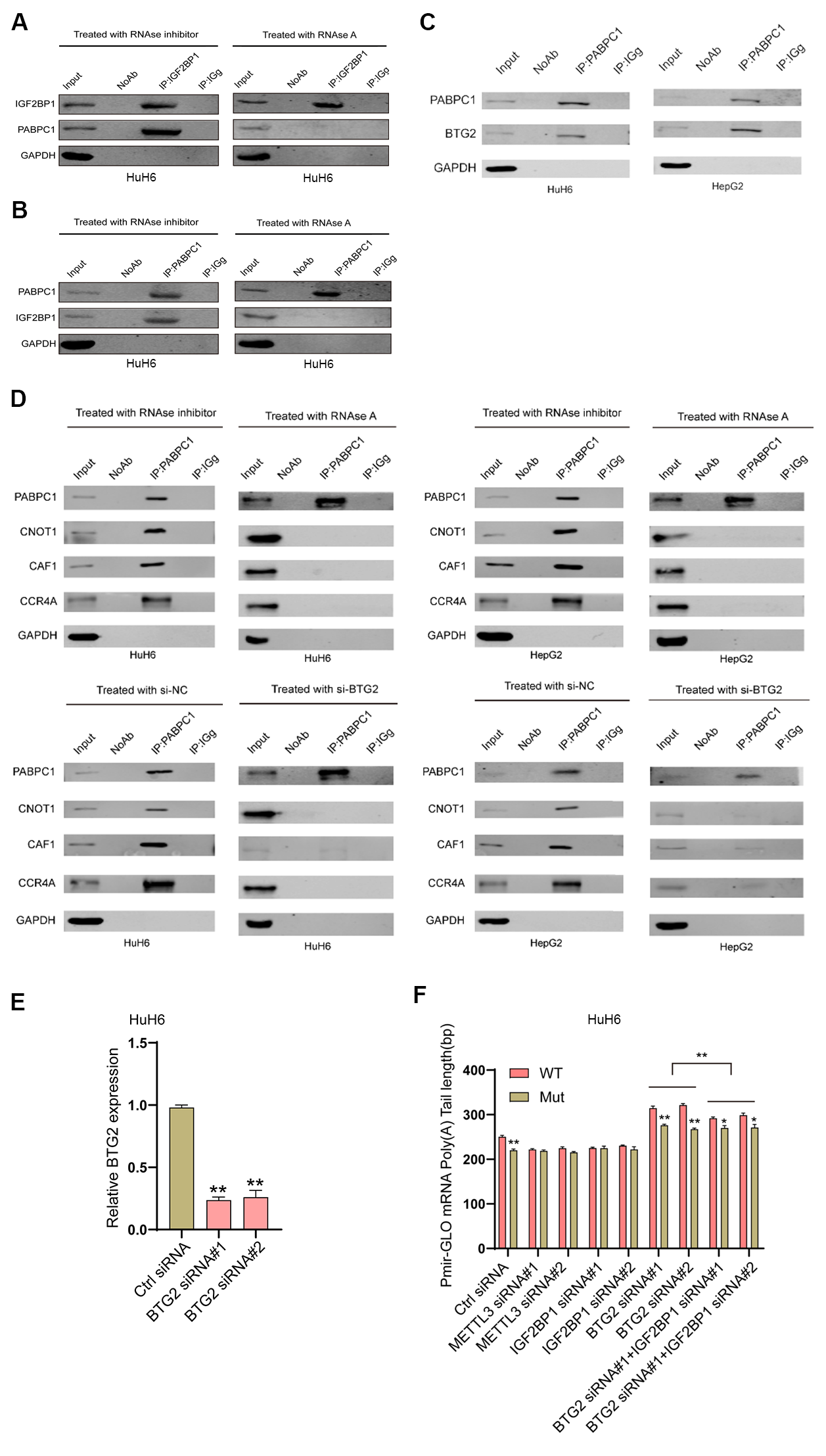

Supplement: Supplementary file 11 — Supporting information. [file CTM2-12-e778-s015.tif]

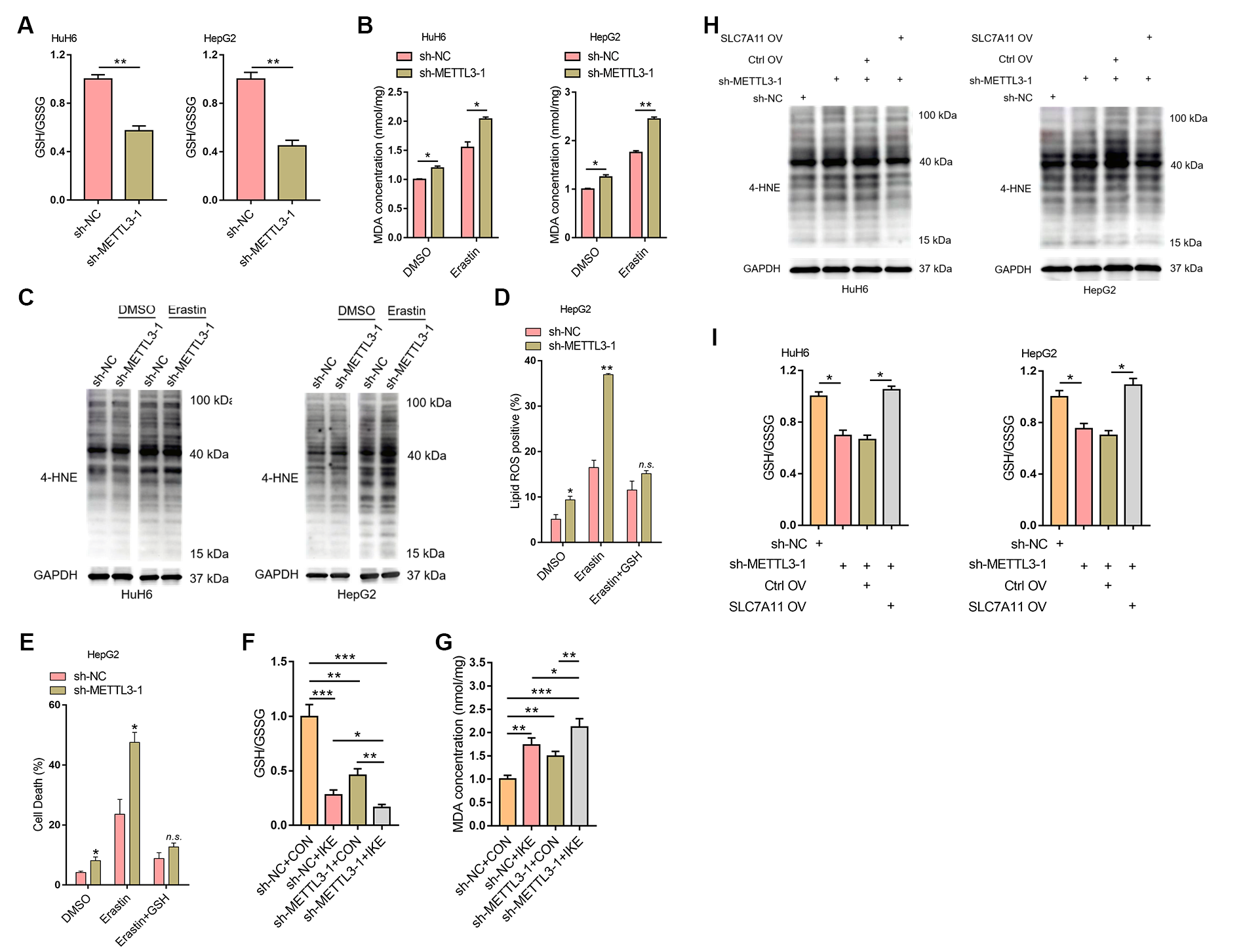

Supplement: Supplementary file 12 — Supporting information. [file CTM2-12-e778-s016.tif]
