# Supplementary material for: The N6‐methyladenosine modification enhances ferroptosis resistance through inhibiting SLC7A11 mRNA deadenylation in hepatoblastoma
Source: Clin Transl Med. 2022 May 6;12(5):e778. doi: 10.1002/ctm2.778 (PMC9076012; doi:10.1002/ctm2.778)
Supplement: Supplementary file 13 — Supporting information. figure‐legends [file CTM2-12-e778-s005.docx]

Supplementary figure legends

Fig. S1. The mRNA expression profile in HB tissues shows that SLC7A11 is a highly expressed gene in HB. A. A heat map summarizes the differentially expressed mRNAs in 5 pairs of tumor and matched normal tissues. Red indicates significantly upregulated mRNAs (fold change > 2.0, p-value < 0.05), and green indicates significantly downregulated mRNAs (fold change < -2.0, p-value < 0.05). B. A scatter plot depicts the expression of mRNAs (log2 (fold change)) in HB tissues vs. normal tissues. The red dots and blue dots represent mRNAs that are significantly overexpressed (log2 (fold change) > 1 and p-value < 0.05) or downregulated (log2 (fold change) < -1 and p-value < 0.05) in tumor tissues compared with normal tissues, respectively. C. A volcano plot constructed with log2 (fold change) and -log10 (p-value) values. The red and blue dots indicate the differentially expressed mRNAs between the two groups. D. KEGG pathway analysis of the upregulated mRNAs. The top ten most significantly enriched terms are exhibited. E. KEGG pathway analysis of the downregulated mRNAs. The top ten most significantly enriched terms are exhibited. F. The expression level (FPKM) of SLC7A11 in 5 pairs of human HB and matched normal tissues according to the mRNA-seq results. G. The protein expression of SLC7A11 in another 7 pairs of human HB and matched normal tissues determined via western blotting. N, normal, T, tumor. **p* < 0.05.

Fig. S2. SLC7A11 promotes proliferation and mediates ferroptosis in HuH6 cells. A-B. HuH6 cells were transfected with siRNAs targeting SLC7A11, and silencing efficiency was verified via RT-qPCR (A) and western blotting (B). C-D. HuH6 cells transfected with SLC7A11 siRNA#1 or SLC7A11 siRNA#2 were subjected to CCK8 assays (C) and colony formation assays (D) to evaluate the role of SLC7A11 in HB cell viability and proliferation. E. HuH6 cells were treated with SLC7A11 siRNA#1 or SLC7A11 siRNA#2 for 36 h, and lipid ROS levels were then measured via BODIPY C11 staining coupled with flow cytometry. F-H. The relative GSH/GSSG ratio (F), 4-HNE (G) and MDA concentration (H) in HuH6 cells, which were transfected with SLC7A11 siRNA#1 or SLC7A11 siRNA#2 for 48h, were detected via GSH/GSSG, western blotting and lipid peroxidation assays, respectively. I. HuH6 cells were treated with SLC7A11 siRNA#1 or SLC7A11 siRNA#2 for 48 h, and cell death was then determined via propidium iodide (PI) staining coupled with flow cytometry. J. The half maximal inhibitory concentration (IC50) of erastin in HepG2 and HuH6 cells. K-L. HuH6 cells transfected with SLC7A11 siRNA#1 or SLC7A11 siRNA#2 were treated with DMSO, ferrostatin-1, erastin, erastin + ferrostatin-1, erastin + ZVAD-FMK, or erastin + necrosulfonamide, respectively, and lipid ROS levels (K) as well as cell death (L) were then determined via flow cytometry. Erastin: 20 μM, Ferrostatin-1: 1 μM, ZVAD-FMK: 10 μM, Necrosulfonamide: 1 μM. Ctrl: control. All quantitative data are shown as the mean ± SD from three independent experiments. *n.s.*, no significant difference, **p* < 0.05, ***p* < 0.01, ****p* < 0.001, *****p* < 0.0001.

Fig. S3. SLC7A11 overexpression promotes HB cell proliferation and enhances HB cell ferroptosis resistance. A-B. The expression of SLC7A11 was determined via RT-qPCR (A) and western blotting (B) in HepG2 and HuH6 cells transfected with SLC7A11 overexpression vector (SLC7A11 OV) or corresponding control vector (Ctrl OV). C-D. The proliferation ability of HepG2 and HuH6 cells upon SLC7A11 overexpression was measured via CCK8 assays (C) and colony formation assays (D). E-F. The levels of lipid ROS (E) and cell death (F) in HepG2 and HuH6 cells transfected with the SLC7A11 overexpression vector were measured via flow cytometry. All quantitative data are shown as the mean ± SD from three independent experiments. *n.s.*, no significant difference, ***p* < 0.01, ****p* < 0.001.

Fig. S4. SLC7A11 promotes HB tumor growth and mediates HB ferroptosis *in vivo*. A. The knockdown efficiency of SLC7A11 administrated with sh-NC, sh-SLC7A11-1, and sh-SLC7A11-2 in HuH6 cells was determined via RT-qPCR. B-D. SLC7A11 deficiency inhibited the subcutaneous tumor growth. Tumor images of the resected sh-NC or sh-SLC7A11 tumors (B). Tumor volumes (mm^3^) were used to draw the tumor growth curves. The tumor volumes were measured using an electronic caliper every 2 days and calculated using the formula volume (mm^3^) = Length (mm)×Width^2^ (mm^2^)/2 (C). Tumor weights of the nude mice injected with shNC, sh-SLC7A11-1, or sh-SLC7A11-2 HuH6 cells were measured after the mice were euthanized at indicated weeks (D). E-F. The relative GSH/GSSG ratio (E) and MDA concentration (F) in xenograft tumors were detected using GSH, GSSG and lipid peroxidation assay kits. G-I. SLC7A11 deficiency enhances HB ferroptosis sensitivity. Tumor images (G), tumor volumes (H) and tumor weights (I) of the xenograft tumors administrated with sh-NC + CON, sh-NC + IKE, sh-SLC7A11-1 + CON, or sh-SLC7A11-1 + IKE. CON, control. **p* < 0.05, ***p* < 0.01, and ****p* < 0.001, *****p* < 0.0001.

Fig. S5. SLC7A11 mRNA was modified by m6A methylation. A. A Venn diagram showing the number of genes (fold change < -2 and > 2, p < 0.05) detected from mRNA-seq and MeRIP-seq in human HB tissues compared to normal tissues. B. Distribution of the overlapping genes with a significant change in both m6A modification and gene expression. SLC7A11 was one of the top overlapping genes with high m6A modification and increased mRNA expression identified in these two sets of data. C. The m6A peaks within SLC7A11 mRNA revealed from MeRIP-seq.

Fig. S6. YTHDF2, YTHDF3, or YTHDC2 depletion cannot influence the expression of SCL7A11. A. The silencing efficiency of IGF2BP2, IGF2BP3, YTHDF2, YTHDF3, and YTHDC2 were verified via RT-qPCR in HuH6 cells. B. Relative expression of SCL7A11 mRNA upon YTHDF2, YTHDF3, or YTHDC2 silencing. Ctrl: control. All quantitative data are presented as the means ± SD of three independent experiments. *n.s.*, no significant difference, ***p* < 0.01.

Fig. S7. The CCR4-NOT complex mediates the deadenylation of SLC7A11 mRNA. A. The silencing efficiency of PAN2, PAN3, PARN, and CNOT1 were verified via RT-qPCR in HuH6 cells. B. The poly(A) tail length of endogenous SLC7A11 transcript upon deadenylase deletion was measured via RACE-PAT assay in HuH6 cells. Ctrl: control. All quantitative data are presented as the means ± SD of three independent experiments. ***p* < 0.01.

Fig. S8. Verification of overexpression efficiency. A. The expression level of IGF2BP1, PARN, PAN2, PAN3, CAF1, CCR4A, CNOT1, and BTG2 were determined via RT-qPCR in HuH6 cells transfected with corresponding overexpression or control vectors. Ctrl: control, OV: overexpression vector. All quantitative data are presented as the means ± SD of three independent experiments. ***p* < 0.01.

Fig. S9. Verification of the interaction between PABPC1 and IGF2BP1, BTG2, CNTO1, CAF1, and CCR4A. A. PABPC1 was co-immunoprecipitated with IGF2BP1 in an RNA-dependent manner in HuH6 cells. B. IGF2BP1 was co-immunoprecipitated with PABPC1 in an RNA dependent manner in HuH6 cells. C. BTG2 was co-immunoprecipitated with PABPC1 in HuH6 and HepG2 cells. D. CNTO1, CAF1, and CCR4A were co-immunoprecipitated with PABPC1 in an RNA- and BTG2-dependent manner in HuH6 and HepG2 cells. E. The silencing efficiency of BTG2 was verified via RT-qPCR in HuH6 cells. F. The poly(A) tail length of pmir-GLO mRNA upon METTL3 deletion, IGF2BP1 deletion, BTG2 deletion, or BTG2 + IGF2BP1 double knockdown was measured via RACE-PAT in HuH6 cells. IP: immunoprecipitation, Ctrl: control, WT: wide type, Mut: mutation. All quantitative data are presented as the means ± SD of three independent experiments. **p* < 0.05, ***p* < 0.01.

Fig. S10. METTL3 deficiency enhances ferroptosis sensitivity in HB cells. A. The relative GSH/GSSG ratio in HuH6 and HepG2 cells upon METTL3 knockdown was detected using GSH, GSSG assay kits. B-C. MDA concentration (B) and 4-HNE (C) in HuH6 and HepG2 cells upon METTL3 knockdown administrated with DMSO, Erastin were determined via western blotting and lipid peroxidation assays. D-E. HuH6 and HepG2 cells stably transfected with sh-METTL3 were treated with DMSO, erastin, or erastin + GSH, respectively, and the levels of lipid ROS (D) and cell death (E) were then measured via flow cytometry. F-G. The relative GSH/GSSG ratio (F) and MDA concentration (G) in xenograft tumors were detected using GSH, GSSG and lipid peroxidation assay kits. H-I. 4-HNE (H) and the relative GSH/GSSG ratio (I) of the HuH6 and HepG2 cells stably transfected with sh-METTL3 upon SLC7A11 overexpression were measured via western blotting and GSH/GSSG assays. Erastin: HepG2 (30 μM) and HuH6 (20 μM), GSH: 0.8 mM. Ctrl: control, OV: overexpression vector. All quantitative data are presented as the means ± SD of three independent experiments. *n.s.*, no significant difference, **p* < 0.05, ***p* < 0.01, ****p* < 0.001.
